# Heterobimetallic multi-site concerted proton electron transfer (MS-CPET) promotes coordination-induced O–H bond weakening†

**DOI:** 10.1039/d5sc03298a

**Published:** 2025-06-05

**Authors:** Julia Feresin, Brett A. Barden, Jayden A. Reyes, Preshit C. Abhyankar, Seth M. Barrett, Christine M. Thomas

**Affiliations:** a Department of Chemistry & Biochemistry, The Ohio State University 100 W.18th Ave Columbus OH 43210 USA thomasc@chemistry.ohio-state.edu; b Department of Chemistry, Muskingum University 260 Stadium Drive New Concord OH 43762 USA sbarrett@muskingum.edu

## Abstract

Coordination-induced bond weakening of X–H bonds (X = O, N, C) has been observed in a number of low-valent transition metal compounds. However, the impact of an appended electron reservoir on the bond dissociation free energy of the O–H bond (BDFE_O–H_) of a substrate bound to a d^0^ metal is poorly understood. To gain insight into the ability of separated deprotonation and oxidation sites to decrease the BDFE_O–H_ during proton-coupled electron transfer (PCET) reactions, a bimetallic system in which the sites of proton and electron loss are two distinct metal sites is described. Herein, the interconversion of tris(phosphinoamide) Zr/Co complexes HO–Zr(MesNP^i^Pr_2_)_3_CoCN^*t*^Bu and O≡Zr(MesNP^i^Pr_2_)_3_CoCN^*t*^Bu *via* hydrogen atom addition/abstraction was studied. Since the Zr center remains in the d^0^ Zr^IV^ state throughout these transformations, the electron transfer process is mediated by the appended redox-active Co^0/I^ center. A series of open-circuit potential (OCP) measurements on the HO–Zr(MesNP^i^Pr_2_)_3_CoCN^*t*^Bu and O≡Zr(MesNP^i^Pr_2_)_3_CoCN^*t*^Bu complexes was performed, from which the BDFE_O–H_ was found to be 64 ± 1 kcal mol^−1^. The BDFE_O–H_ value was further verified through a series of stoichiometric H atom transfer reactions, stoichiometric protonation/deprotonation reactions, and computational studies.

## Abstract 2

Appending a redox-active Co atom to a redox-inactive d^0^ Zr^IV^ center is shown to weaken the O–H bond of a bound hydroxide ligand in an example of multi-site concerted proton electron transfer (MS-CPET).

## Graphical abstract

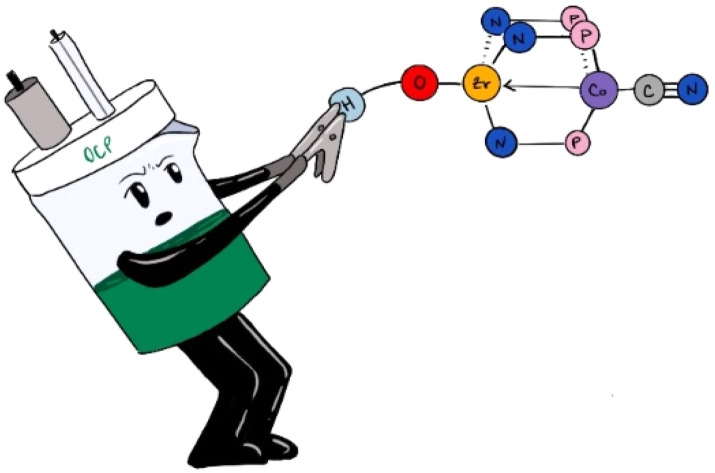

## Introduction

**Fig. 1:**
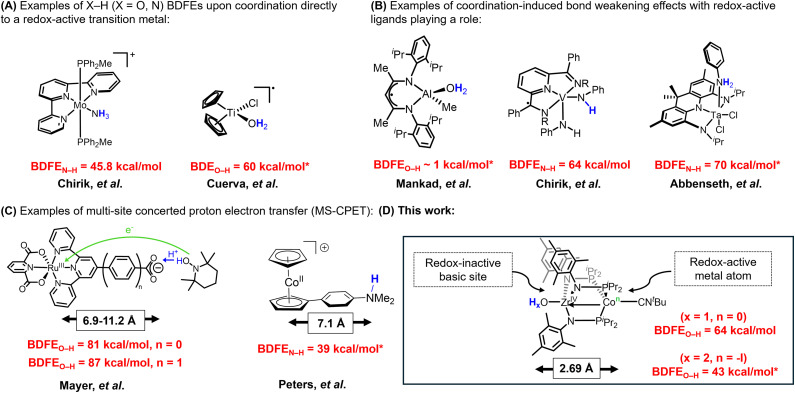
(A) Examples of X–H (X = O, N) BDFEs upon coordination to a redox-active transition metal, (B) examples of coordination-induced bond weakening effects with redox-active ligands playing a role, (C) examples of multi-site concerted proton electron transfer (MS-CPET), and (D) this work. BDFE values with * corresponds to DFT calculated values.

Coordination-induced bond weakening describes the diminished energy required to homolytically cleave the element-hydrogen (X–H; X = O, N, C) bonds of a metal-bound substrate. Homolytic bond cleavage typically involves one-electron oxidation of the coordinating metal complex in a proton-coupled electron transfer (PCET) reaction.^1^ Coordination-induced bond weakening has been shown to promote the dehydrogenation of feedstock molecules such as water and ammonia, reactions that have potential applications in renewable chemical fuel storage systems.^1^ Chirik and coworkers reported an example of a redox-active molybdenum complex that lowered the bond dissociation free energy of the N–H bonds in ammonia (BDFE_N–H_) to 45.8 kcal mol^−1^ (Fig. 1A), suggesting potential applications of coordination-induced bond weakening in the fields of catalysis, bioinorganic chemistry, and alternative energy.^2^ However, the first example of coordination-induced bond weakening in early transition metals was observed by Cuerva *et al.* with a titanocene(iii) system that mediated radical reductions using water as a hydrogen-atom source (Fig. 1A).^3^ Coordination-induced bond weakening has also been demonstrated with main group compounds and transition metal clusters.^4,5^ Although coordination-induced bond weakening has been established for transition metal compounds, accessing the requisite low-valent state can be challenging. Thus, redox-active ligands have been employed as a strategy to promote coordination-induced bond weakening of substrates bound to redox-inactive metals, as examined by Mankad and coworkers using an aluminum-containing compound (Fig. 1B).^6^ A report from Chirik *et al.* subsequently demonstrated the potential for redox-active ligands to serve as electron-reservoirs for N–H bond cleavage/formation in vanadium amido/imido complexes using redox-active pyridine diimine ligands (Fig. 1B).^7^ More recently, the Abbenseth group demonstrated the weakening of aniline and anilide N–H bonds in a series of d^0^ Ta^V^ compounds ligated by a redox-active NNN pincer ligand (Fig. 1B).^8^ The use of redox-active ligands spawns a different subset of PCET reactions, namely multi-site concerted proton electron transfer (MS-CPET), a term used to describe a process where the proton and electron originate and terminate at different sites within a molecule.^9,10^

MS-CPET mechanisms are employed in a variety of biological systems.^11–20^ Understanding the relationship between separated electron transfer (ET) and proton transfer (PT) sites promotes deeper insight into the mechanisms by which MS-CPET enables reactions that would otherwise be thermodynamically unfavorable.^9,10^ Thus, studying the impacts of extensive separation of proton and electron donor/acceptor sites in well-defined molecular species can help clarify the roles of these sites in complex biological or materials-based systems. The Mayer group previously reported examples of MS-CPET mechanisms in molecular transition metal model systems.^21–26^

For example, they measured the BDFE_O–H_ of a carboxylic acid functionality appended to a Ru-bound terpyridine ligand and the impact of increasing the separation between proton (O–H) and electron (Ru) transfer sites by inserting a phenyl ring between the terpyridine ligand and the carboxylate fragment, ultimately finding the O–H bond to be weakened by the appended redox-active Ru center even with a separation of 11.2 Å between the proton and electron transfer sites (Fig. 1C).^26^ In related work, Peters and coworkers have leveraged the reducing nature of the cobaltacene fragment to generate potent H-atom transfer reagents, where the H^+^ originates at an ammonium site more than 7 Å away from the redox-active cobalt center (Fig. 1C).^27^ Herein, we set out to explore the impact of MS-CPET in a multimetallic system in which the proton and electron transfer steps occur at different metal centers and the extent to which coordination-induced bond weakening can be realized in substrates bound to a d^0^ metal center with the aid of a pendent redox-active metal atom (Fig. 1D).

**Scheme 1:**
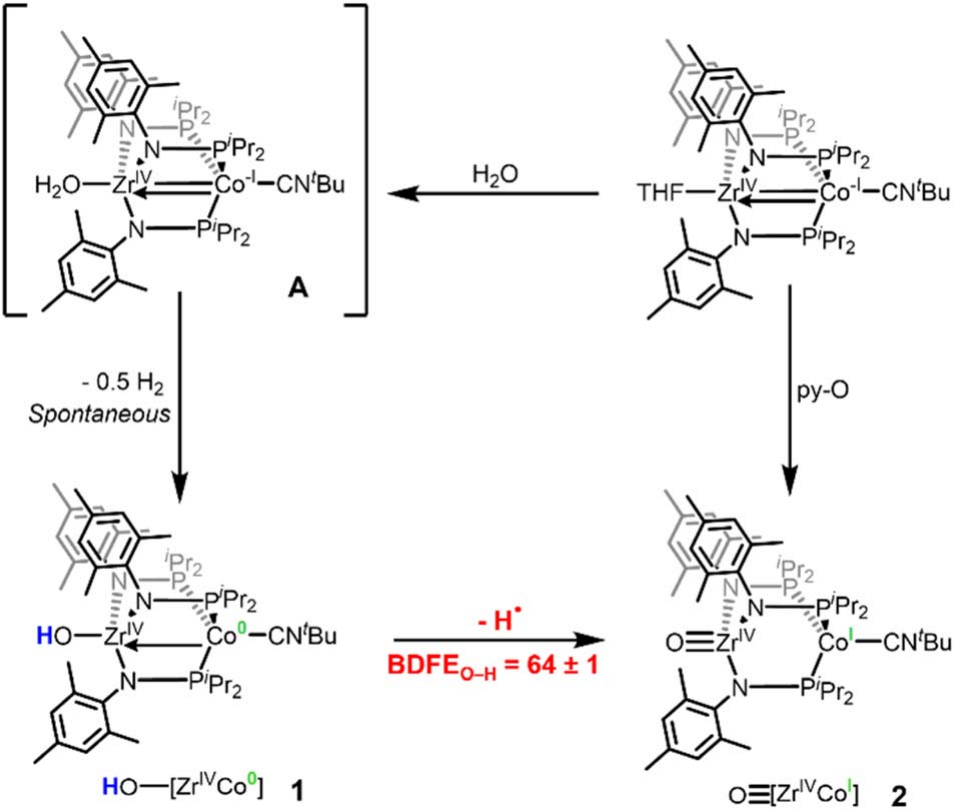
Reported synthetic procedures to generate 1 and 2 and sequential H-atom removal from Zr-OH_*x*_ (*x* = 2, 1) fragments in a Zr/Co heterobimetallic system.

During the course of our previous studies on metal–metal cooperativity in early/late heterobimetallic compounds,^28^ we described a tris(phosphinoamide) Zr^IV^/Co^−I^ complex, (THF)Zr(MesNP^i^Pr_2_)_3_CoCN^*t*^Bu, in which a redox-active Co^−I^ center is appended to a d^0^ Zr^IV^ center.^29^ By sterically blocking the Co site with a tightly binding ^*t*^BuNC ligand, substrate binding can only occur at the redox-inactive Zr site and Co plays the role of an electron reservoir. This strategy permitted oxidative group transfer at the formally d^0^ Zr center to generate a terminal Zr-imido compound and two-electron reduction of O_2_ to generate an η^2^-peroxo compound.^29,30^ The addition of one equivalent of water to (THF)Zr(MesNP^i^Pr_2_)_3_CoCN^*t*^Bu afforded a transient intermediate H_2_O–Zr(MesNP^i^Pr_2_)_3_CoCN^*t*^Bu (A) with a sufficiently low BDFE_O–H_ that H_2_ is spontaneously released to afford the Zr^IV^/Co^0^ hydroxide compound HO–Zr(MesNP^i^Pr_2_)_3_CoCN^*t*^Bu (1).^31^ Treatment of (THF)Zr(MesNP^i^Pr_2_)_3_CoCN^*t*^Bu with pyridine-N-oxide (py-O) afforded the Zr^IV^/Co^I^ oxo species O≡Zr(MesNP^i^Pr_2_)_3_CoCN^*t*^Bu (2) (Scheme 1).^30^ Hydrogen atom abstraction from 1 to generate 2 would rely on loss of a proton from the Zr-bound hydroxide ligand and an electron from the Co center, sites that are separated by 2.69 Å (Scheme 1). Herein, we report the first example, to our knowledge, of a MS-CPET process in which the electron transfer and the proton transfer steps occur at two different metal centers, allowing a d^0^ metal to undergo a PCET reaction. To better examine the impact of separated oxidation and deprotonation sites on the coordination-induced weakening of the O–H bond, we measure the O–H bond dissociation free energy (BDFE_O–H_) within compound 1.

## Results & discussion

Compounds 1 and 2 were synthesized using reported procedures,^30,31^ followed by electrochemical experiments to determine the BDFE_O–H_ of the hydroxide ligand in 1. In 2020, the Mayer group reported a method to calculate the BDFE of polar X–H bonds (O–H and N–H) in nonaqueous solvents using open-circuit potential (OCP) measurements of a 1 : 1 mixture of the two compounds that differ by one H atom in a buffered electrolyte solution.^32^ The 
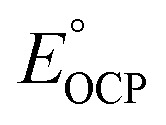
 values are referenced against the H^+^/H_2_ couple and the BDFE_X–H_ can be calculated using eqn (1).^32–36^ The 
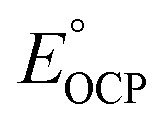
 is measured using a solution containing equal concentrations of both the oxidized and reduced species (X/XH) and 
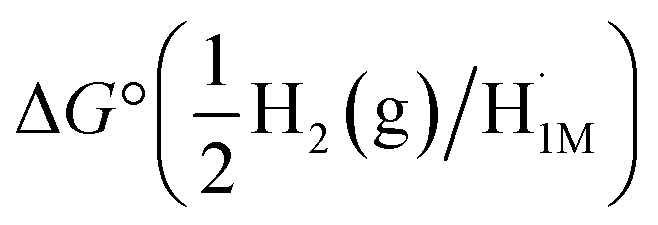
 is a solvent-related constant describing the free energy of H_2_ homolytic cleavage.^32^1
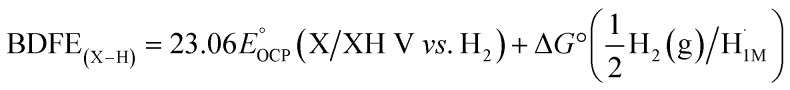


To obtain the BDFE_O–H_ of 1 using OCP measurements, a series of buffered electrolyte solutions in THF (100 mM [^*n*^Bu_4_N][PF_6_], 50 mM lutidine (lut), and 50 mM [Hlut][BPh_4_]) containing both the oxidized (2) and reduced (1) species were prepared. OCP measurements (referenced to the ferrocenium/ferrocene redox couple, Fc^+/0^) were collected using five different ratios of hydroxide (1): oxo (2) to plot the OCP *vs.* log([1]/[2]) and find the *y*-intercept that represents the 
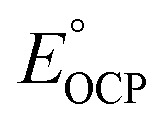
 of the 1 : 1 ratio between the oxidized (2) and reduced (1) species needed in eqn (1). The OCP measurements of five varying ratios of 1 and 2 also provide insight into whether the MS-CPET behaves as an ideal, Nernstian system (eqn (2)).^32^ Three overall trials at each hydroxide (1) : oxo (2) ratio were performed to confirm reproducibility (ESI Section 2.2†).2
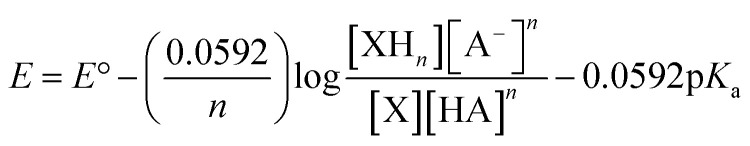


**Fig. 2:**
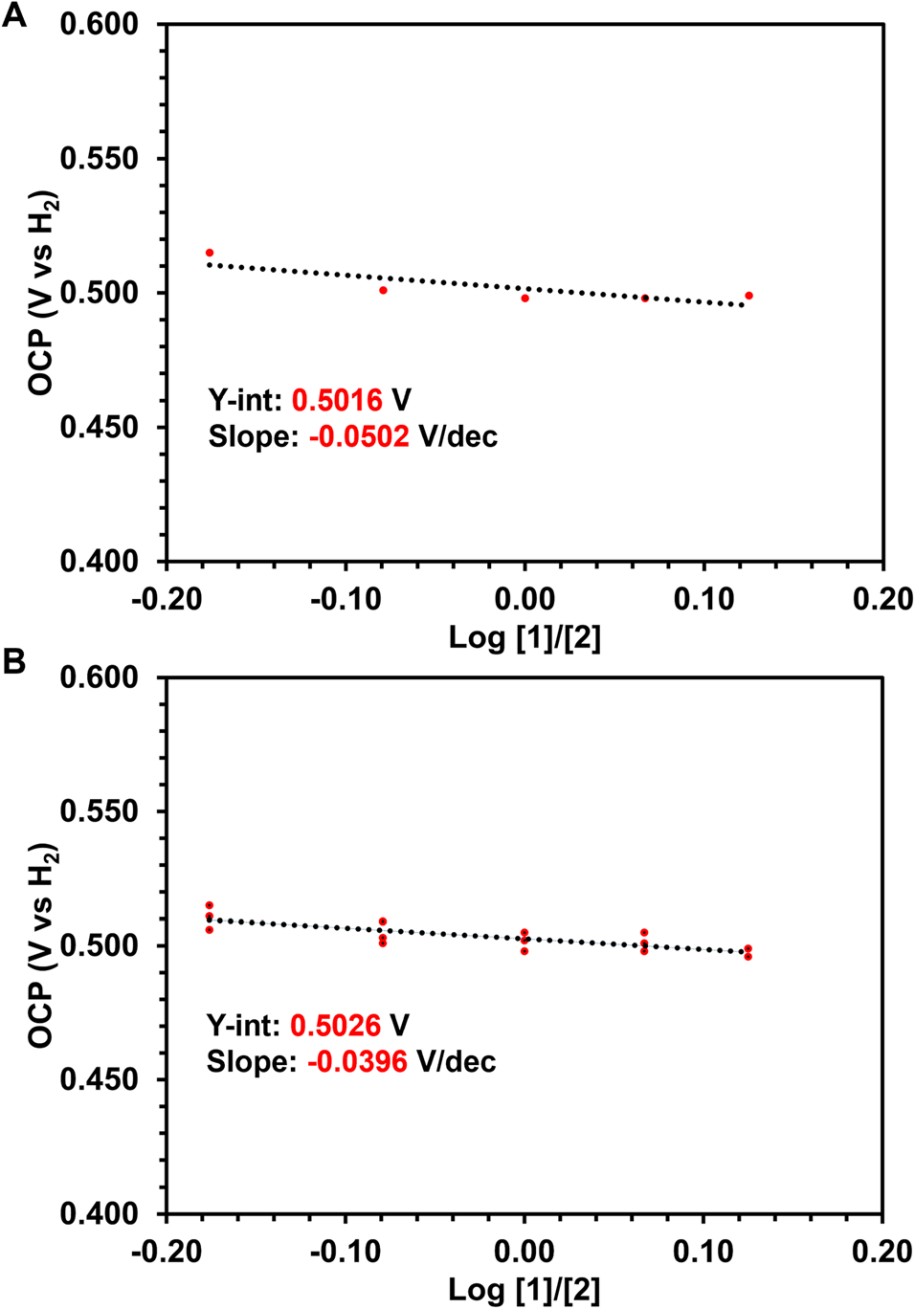
(A) Representative trial of the OCP (V *vs.* H_2_) *vs.* the log of varying ratios of 1 and 2. (B) OCP (V *vs.* H_2_) *vs.* the log of varying ratios of 1 and 2 for all three trials.

The measured OCP was then referenced to the H^+^/H_2_ couple in the same buffered solution (100 mM [^*n*^Bu_4_N][PF_6_], 50 mM lutidine, and 50 mM [Hlut][BPh_4_] in THF) in the presence of H_2_ (ESI, Section 2.1†). The 
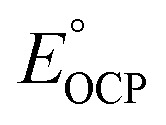
 (H^+^/H_2_) was referenced *vs.* Fc^+/0^, allowing the OCP measurements of each hydroxide (1) : oxo (2) ratio to be referenced to H^+^/H_2_ prior to plotting the OCP (V *vs.* H_2_) *vs.* log [1]/[2] (Fig. 2). Fig. 2A shows an example of one of the three trials performed, in which the *y*-intercept provides information about the 
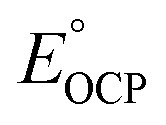
 (1/2) at the 1 : 1 ratio. The average *y*-intercept for the three trials was found to be 0.503 V *vs.* H_2_. When all three trials are plotted together (Fig. 2B), minimal deviation in the OCP is observed between trials, yielding a consistent OCP value within 5 mV. Furthermore, the slope of the OCP (V *vs.* H_2_) *vs.* log [1]/[2] plot provides information about the behavior of the system. In an ideal system for a one-electron process, the OCP should decrease by 0.0592 V dec^−1^ for each order of magnitude change in ratio between hydroxide and oxo, as described by eqn (2).^32^ The slopes obtained in all three trials ranged from −0.0274 V dec^−1^ to −0.0502 V dec^−1^, which is in reasonable agreement with ideal Nernstian behavior considering the use of a low dielectric constant non-aqueous solvent (THF).

By referencing the OCP of the equimolar mixture of 1 and 2 to the OCP of the H^+^/H_2_ solution, a direct route was used to calculate the BDFE_O–H_.^32^ By substituting the 
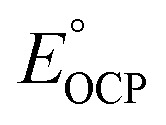
 (V *vs.* H_2_), which was found to be 0.503 V *vs.* H_2_, into eqn (1), the BDFE_O–H_ obtained was 63.6 (64 ± 1) kcal mol^−1^, since the value for the 
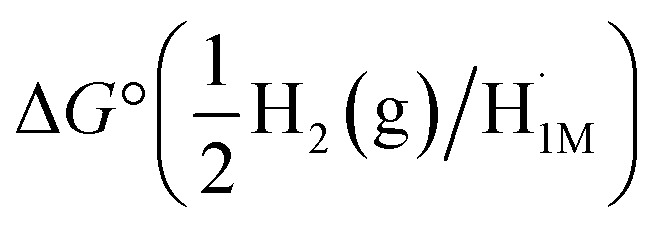
 is known to be 52.0 kcal mol^−1^ in THF.^32^ For further sample calculations and error analysis, consult ESI Sections 3.1 and 3.3,† respectively.

**Scheme 2:**
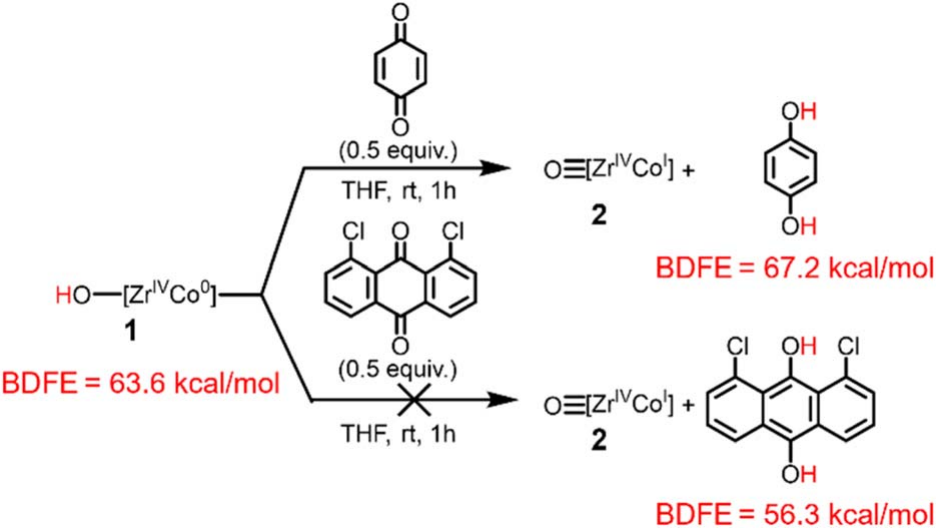
Reactivity of complex 1 towards H-atom abstraction reagents.

To provide further support for the BDFE_O–H_ value of the hydroxide compound 1 determined *via* the OCP measurements, the stoichiometric reactivity of 1 towards H atom abstractors was investigated (Scheme 2). Further reactivity studies of 1 with additional H atom abstractors and of oxo (2) towards H atom donors is described in the ESI, Section 4.† When complex 1 (BDFE_O–H_ = 63.6 kcal mol^−1^) was treated with 2,4,6-tri-*tert*-butylphenoxyl radical (BDFE_O–H_ = 74.4 kcal mol^−1^)^37^ generation of 2 was observed (ESI, Section 4.1†). Moreover, when 2 was treated with 9,10-dihydroanthracene (BDFE_C–H_ = 72.9 kcal mol^−1^)^37^ no reaction occurred (ESI, Section 4.2†), confirming that the BDFE_O–H_ must be less than 73 kcal mol^−1^. Treatment of complex 1 with *p*-benzoquinone (BDFE_O–H_ = 67.2 kcal mol^−1^)^37^ resulted in the formation of 2, but no reactivity was observed between 1 and 1,8-dichloroanthraquinone (BDFE_O–H_ = 56.3 kcal mol^−1^)^37^ (Scheme 2). Thus, this series of H atom transfer reactions provides a BDFE_O–H_ range of 67.2 > BDFE_O–H_ > 56.3, which agrees with the BDFE_O–H_ = 64 ± 1 determined *via* the OCP measurements.

**Scheme 3:**
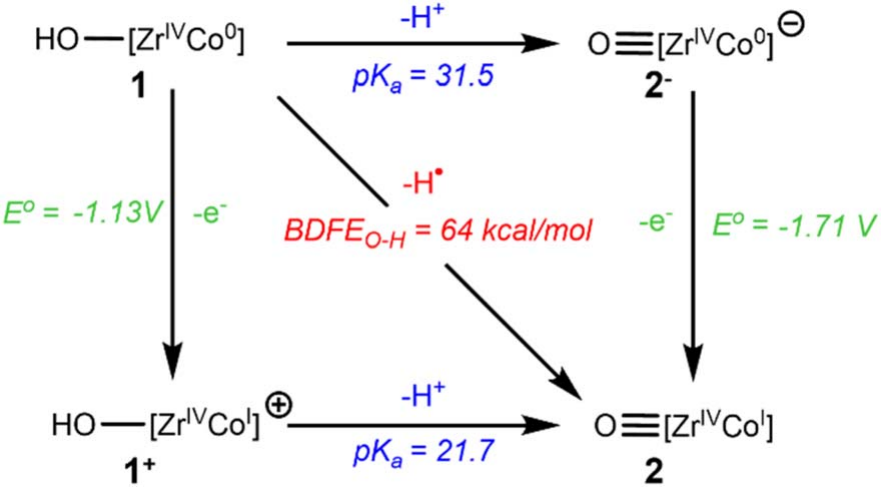
Square scheme showing the stepwise and concerted interconversion of 1 and 2. The BDFE_O–H_ and *E*° values were experimentally determined using OCP and CV measurements^[31]^ and the p*K*_a_ values are estimated from the BDFE_O–H_ and *E°* values using eqn (3).

Following experimental verification of the BDFE_O–H_ value determined *via* OCP measurements, the p*K*_a_ values of the neutral hydroxide complex 1 and the cationic hydroxide complex [HO–Zr(MesNP^i^Pr_2_)_3_CoCN^*t*^Bu]^+^ (1^+^) could be estimated using the square scheme shown in Scheme 3, the Bordwell equation (eqn (3)),^38^ the established solvent-specific constant (*C*_g,sol_ = 59.9 kcal mol^−1^) for THF,^37^ and the previously reported redox potentials for complexes 1 and 2 determined *via* cyclic voltammetry (CV)^30,31^ (ESI, Section 3.2†). The p*K*_a_ values for complexes 1 and 1^+^ were calculated to be 31.5 and 21.7, respectively (Scheme 3). It is important to note that the p*K*_a_ values calculated using this method represent the p*K*_a_ values in a buffered electrolyte solution. As such, the calculated p*K*_a_ values can be considered estimates, not exact values.

3 BDFE_(X−H)_ = 23.06*E*°(X^0/−^) + 1.37p*K*_a_(X−H) + *C*_g,sol_

**Scheme 4:**
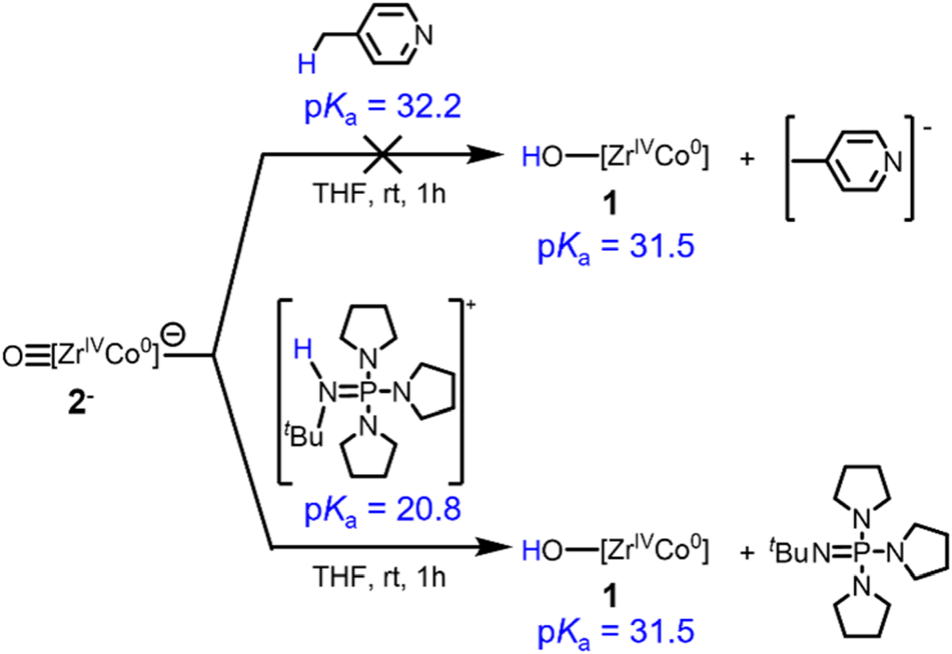
Reactivity of complex 2^−^ with different acids to estimate the upper and lower bounds of the p*K*_a_ of complex 1.

To provide support for the estimated p*K*_a_ values and further verify the BDFE_O–H_ determined *via* OCP measurements, the reactivity of previously reported anionic oxo complex [OZr(MesNP^i^Pr_2_)_3_CoCN^*t*^Bu]^−^ (2^−^) toward acids was investigated (Scheme 4). The estimated p*K*_a_ value for 1 of 31.5 is consistent with the observed lack of reaction between 2^−^ and 4-methylpyridine (p*K*_a_ = 32.2).^39^ In contrast, 2^−^ was found to react with [^*t*^BuHN=P(pyrr)][BPh_4_] (pyrr = pyrrolidinyl, p*K*_a_ = 20.8)^40^ to generate 1. Thus, the stoichiometric protonation/deprotonation reactions provided a p*K*_a_ range for complex 1 of 32.2 > p*K*_a_ > 20.8, which agrees with the estimated value of 31.5.

**Scheme 5:**
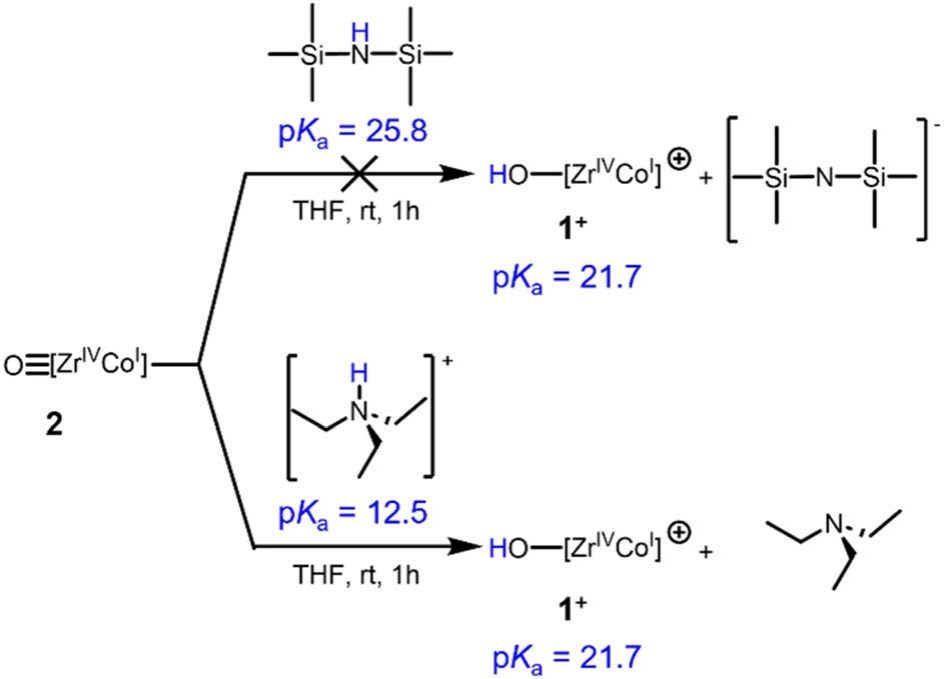
Reactivity of complex 2 with different acids to estimate the upper and lower bounds of the p*K*_a_ of complex 1^+^.

To verify the estimated p*K*_a_ value of 21.7 for the cationic hydroxide compound 1^+^, the protonation of 2 was investigated (Scheme 5). No reaction occurs between complex 2 and (Me_3_Si)_2_NH (p*K*_a_ = 25.8).^39^ Oxo complex 2 was, however, readily protonated with [HNEt_3_][BPh_4_] (p*K*_a_ = 12.5)^40^ to afford the previously reported cationic hydroxide compound 1^+^. The p*K*_a_ range for the cationic hydroxide 1^+^ was therefore experimentally determined to be 25.8 > p*K*_a_ > 12.5, which agrees with the estimated p*K*_a_ value of 21.7.

After further verification of the BDFE_O–H_ value *via* BDFE and p*K*_a_ test reactions, density functional theory (DFT) was used to compute BDFE and p*K*_a_ values to provide additional support. Eight H atom donors were used to construct a BDFE calibration curve using known BDFE values and computed free energy values (ESI Section 6.3†), yielding a calculated BDFE_O–H_ value of 60 ± 4 kcal mol^−1^ for the hydroxide complex 1. The calculated and experimental BDFE_O–H_ are within error of one another. Thus, the same computational method was used to calculate the BDFE_O–H_ for the unobservable H_2_O-bound transient intermediate complex A, yielding a BDFE_O–H_ of 42.9 ± 4 kcal mol^−1^. This value is consistent with the spontaneous loss of H˙ and formation of H_2_ (BDFE_H–H_ = 104 kcal mol^−1^),^41^ which is presumed to proceed *via* a bimolecular mechanism. The p*K*_a_ values of 1 and 1^+^ were also computed using DFT. Nine organic acids were used to construct a p*K*_a_ calibration curve using known p*K*_a_ values and computed free energy values (ESI Section 6.4†), yielding a p*K*_a_ value of 27.9 for the neutral hydroxide 1 and 21.8 for the cationic hydroxide 1^+^. The computed BDFE_O–H_ and p*K*_a_ values agree well with experimentally determined values.

## Conclusions

In summary, the analysis of OCP measurements referenced to H^+^/H_2_ has been shown to be a facile technique for determining the BDFE of the terminal hydroxide functionality of the Zr/Co complex 1. It was found that the O–H bond in complex 1 has a BDFE of 64 ± 1 kcal mol^−1^, and this value has been supported by both DFT computations and reactions with stoichiometric H-atom transfer reagents. Using the BDFE_O–H_ value of 64 ± 1 kcal mol^−1^ and a square scheme analysis, the p*K*_a_ of complexes 1 and 1^+^ were estimated to be 31.5 and 21.7, respectively.

Through this study, it can be concluded that in both Zr–OH and Zr–OH_2_ compounds, the BDFE_O–H_ is dramatically decreased by the presence of an appended redox-active metal center. Importantly, this demonstrates the viability of the multimetallic system to facilitate element–hydrogen bond cleavage, as significant coordination-induced bond weaking was observed despite the separation between the proton and electron transfer sites: Although the d^0^ Zr^IV^ center to which the hydroxide ligand is directly bound is redox-inactive, the electron-transfer capacity of the appended Co center in complex 1 results in a similar degree of coordination-induced bond weakening as would be expected if the substrate were directly bound to a redox-active metal. The low BDFE_O–H_ value of 64 ± 1 kcal mol^−1^ within HO–Zr^IV^/Co^0^ complex 1 is at the low end of the range of BDFE_O–H_ values reported for terminal Co^II^, Fe^III^, Fe^II^, and Mn^II^ hydroxide compounds (64–85 kcal mol^−1^).^42–44^ Although the corresponding H_2_O–Zr^IV^/Co^−I^ compound A cannot be isolated, the spontaneous release of H_2_ from this aquo intermediate suggests a BDFE_O–H_ value lower than half the BDFE_H–H_ of H_2_ (52 kcal mol^−1^), which is substantiated by a DFT-calculated BDFE_O–H_ of 43 kcal mol^−1^. This represents a coordination-induced weakening of the O–H bond of ∼70 kcal mol^−1^ when compared to the BDFE_O–H_ of free H_2_O (BDFE = 115.8 kcal mol^−1^).^37^ The extent of coordination-induced O–H bond weakening in 1 and A is similar to the effect expected if the H_2_O and OH^−^ ligands were directly bound to a redox-active metal, demonstrating that a redox-active metal appended to the substrate binding site is a viable strategy to facilitate element–hydrogen bond activation. The estimated p*K*_a_ values for 1 and 1^+^ are unremarkable compared to monometallic terminal hydroxide compounds^45^ indicating that the weakening of the O–H bond is, indeed, driven by the low Co^I/0^ redox potential. Future studies will explore whether the coordination-induced bond weakening phenomenon can be generalized across other element-hydrogen bond-containing substrates and bimetallic combinations and seek to establish applications for the resulting H-atom transfer processes.

## Author contributions

C. M. T. supervised and acquired funding for the project. J. F., S. M. B., and J. A. R. conducted the experiments and data analysis. B. A. B. and C. M. T. conceptualized the goals and aims of the project and B. A. B. collected the preliminary data on which this study is based. P. C. A. performed and analyzed the DFT calculations. J. F., S. M. B., and C. M. T. contributed to writing, reviewing, and editing the manuscript and all authors gave approval to the final version.

## Conflicts of interest

There are no conflicts to declare.

## Supplementary Material

SC-OLF-D5SC03298A-s001

## Data Availability

The data supporting this article have been included as part of the ESI.†
