# Supplementary material for: Heterobimetallic multi-site concerted proton electron transfer (MS-CPET) promotes coordination-induced O–H bond weakening
Source: Chem Sci. 2025 Jun 5;16(28):12941–6. doi: 10.1039/d5sc03298a (PMC12168920; doi:10.1039/d5sc03298a)
Supplement: SC-016-D5SC03298A-s001 [file SC-016-D5SC03298A-s001.pdf]

Supplementary Information (SI) for Chemical Science.
This journal is © The Royal Society of Chemistry 2025

SUPPORTING INFORMATION

Coordination-induced O-H bond weakening in a heterobimetallic Zr/Co complex

Julia Feresin,[a] Brett A. Barden,[a] Jayden A. Reyes,[a] Preshit C. Abhyankar,[a] Seth M. Barrett,[b]* and Christine M. Thomas[a]*

[a]Department of Chemistry and Biochemistry, The Ohio State University, 100 W. 18th Ave, Columbus, OH 43210, USA

[b]Department of Chemistry, Muskingum University, 260 Stadium Drive, New Concord, OH 43762, USA

TABLE OF CONTENTS

1. Synthesis and Characterization Details 2

1.1. General Considerations 2
1.2. Synthetic Procedures 2

2. Cyclic Voltammetry & Open-Circuit Potential 5

2.1. E_{OCP} (H^+/H_2) vs $Fc^{+/0}$ 5
2.2. E_{OCP} (O/OH) vs H_2 8

3. Direct conversion of OCP (V vs H_2) to $BDFE_{O-H}$ 17

3.1. $BDFE_{O-H}$ sample calculation 17
3.2. pK_a sample calculation 18
3.3. Uncertainty in $BDFE_{O–H}$ 18

4. $BDFE_{O–H}$ test reactions 19

4.1. HO–Zr(MesNP^{i}Pr$_2$)$_3$CoCN^{t}Bu (1) with 2,4,6-tri-tert-butylphenoxyl radical 19
4.2. O≡Zr(MesNP^{i}Pr$_2$)$_3$CoCN^{t}Bu (2) with 9,10-dihydroanthracene 21
4.3. HO–Zr(MesNP^{i}Pr$_2$)$_3$CoCN^{t}Bu (1) with *p*-benzoquinone 23
4.4. HO–Zr(MesNP^{i}Pr$_2$)$_3$CoCN^{t}Bu (1) with 1,8-dichloroanthraquinone 24

5. pK_a test reactions 26

5.1. [(μ-Na)OZr(MesNP^{i}Pr$_2$)$_3$CoCN^{t}Bu]$_2$ (2^-) with 4-methylpyridine 26
5.2. [(μ-Na)OZr(MesNP^{i}Pr$_2$)$_3$CoCN^{t}Bu]$_2$ (2^-) with [^{t}BuHP$_1$pyrr][BPh$_4$] 28
5.3. O≡Zr(MesNP^{i}Pr$_2$)$_3$CoCN^{t}Bu (2) with (Me$_3$Si)$_2$NH 30
5.4. O≡Zr(MesNP^{i}Pr$_2$)$_3$CoCN^{t}Bu (2) with [HNEt$_3$][BPh$_4$] 32

6. Computational Details 35

6.1. General computational considerations 35
6.2. Estimating $BDFE_{O-H}$ using a DFT-based BDFE calibration curve 36
6.3. $BDFE_{O-H}$ sample calculation using the DFT BDFE calibration curve 37
6.4. Estimating pK_a using a DFT-based pK_a calibration curve 37
6.5. pK_a sample calculation using the DFT pK_a calibration curve 39

7. References 39

1. Synthesis and Characterization Details

1.1. General Considerations

All experimental manipulations were carried out in a nitrogen-filled glovebox or via standard Schlenk techniques, unless otherwise noted. All glassware was oven-dried for a minimum of two hours prior to use. All protio solvents were degassed with ultra-high purity argon, dried using a Pure Process Technology solvent purification system, and stored over activated 3 Å molecular sieves. Benzene-d_6 was degassed via repeated freeze-pump-thaw cycles using an oven-dried Schlenk flask and dried by storing over activated 3 Å molecular sieves. Lutidine (lut), hexamethyldisilazane (HMDS), and 4-methylpyridine were dried over CaH_2, distilled into an oven-dried vessel, and degassed by repeated freeze-pump-thaw cycles prior to bringing into the glovebox and storage over 3 Å molecular sieves. *p*-Benzoquinone was purchased commercially and purified by sublimation prior to bringing into a nitrogen-filled glovebox. Tetrabutylammonium hexafluorophosphate ([^{n}Bu$_4$N][PF$_6$]) was recrystallized twice from a saturated ethanol solution and dried under vacuum at 60 °C for three days before bringing into a glovebox. All other chemicals, except the reagents and precursors noted in Section 1.2, were purchased from commercial vendors and used without further purification. NMR spectra were recorded at ambient temperature on a Bruker Advance Neo 400 MHz (^{11}B: 128 MHz, ^{31}P: 162 MHz) or a Bruker AVIII 600 MHz instrument. For ^{1}H NMR spectra, the solvent resonance was used as an internal standard. For ^{11}B NMR spectra, boron trifluoride etherate (BF_3•Et_2O) was used as an external standard (0 ppm). For ^{31}P experiments, 85% phosphoric acid (H_3PO_4) was used as an external standard (0 ppm).

1.2. Synthetic Procedures

O≡Zr(MesNP^{i}Pr$_2$)$_3$CoCN^{t}Bu (**2**),[1] HO–Zr(MesNP^{i}Pr$_2$)$_3$CoCN^{t}Bu (**1**),[2] [(μ-Na)OZr(MesNP^{i}Pr$_2$)$_3$CoCN^{t}Bu]$_2$ (**2**$^-$),[2] and the 2,4,6-tri-*tert*-butylphenoxyl radical[3] were synthesized following literature procedures. Lutidinium tetraphenylborate ([Hlut][BPh$_4$]) was synthesized in a fume hood via modification of literature procedures[4,5] by means of salt metathesis of lutidinium chloride and sodium tetraphenylborate in methanol. The solids were filtered, washed

with water and ether, then dried in vacuo at 70 °C for three days prior to bringing into a glovebox. Triethylammonium tetraphenylborate ([$HNEt_3$][BPh_4]) was synthesized in a fume hood via modified literature procedures[4,5] by means of salt metathesis of triethylammonium chloride and sodium tetraphenylborate in water. The solids were filtered, washed with water, and then dried in vacuo at 150 °C for three days prior to bringing into a glovebox. The [^{t}BuHP_1pyrr][BPh_4] (P_1 = P═N and pyrr = tetramethyleneamino) salt was synthesized via a protonation reaction of ^{t}BuP_1pyrr with [Hlut][BPh_4]. ^{1}H NMR (400 MHz, CD_2Cl_2): δ 7.34 – 7.26 (m, 8H), 7.03 (t, J = 7.4 Hz, 8H), 6.87 (td, J = 7.1, 1.6 Hz, 4H), 3.20 – 3.10 (m, 12H), 2.55 (s, 1H), 1.98 – 1.90 (m, 12H), 1.32 (s, 9H). ^{31}P{^{1}H} NMR (162 MHz, CD_2Cl_2): δ 20.65. ^{11}B NMR (128 MHz, CD_2Cl_2) :δ -6.74. The spectroscopy data obtained is consistent with previously reported data.[6]

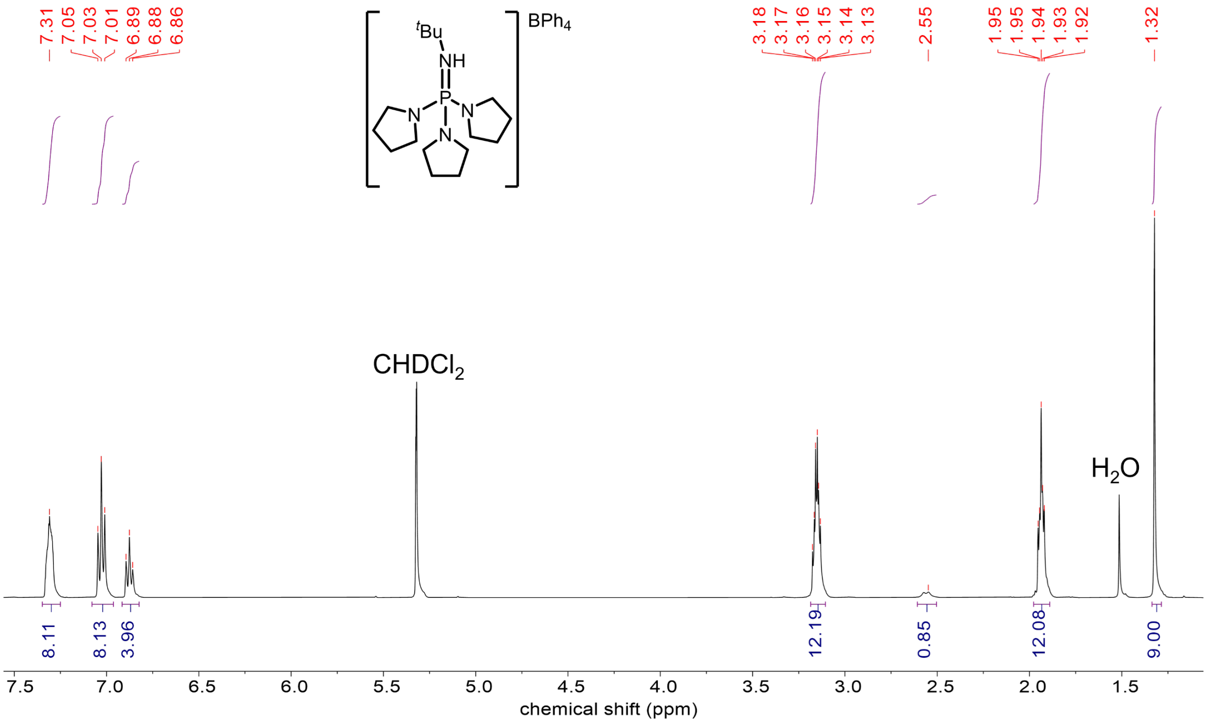

Figure S1. ^{1}H NMR spectrum (400 MHz, CD_2Cl_2) of [^{t}BuHP_1pyrr][BPh_4].

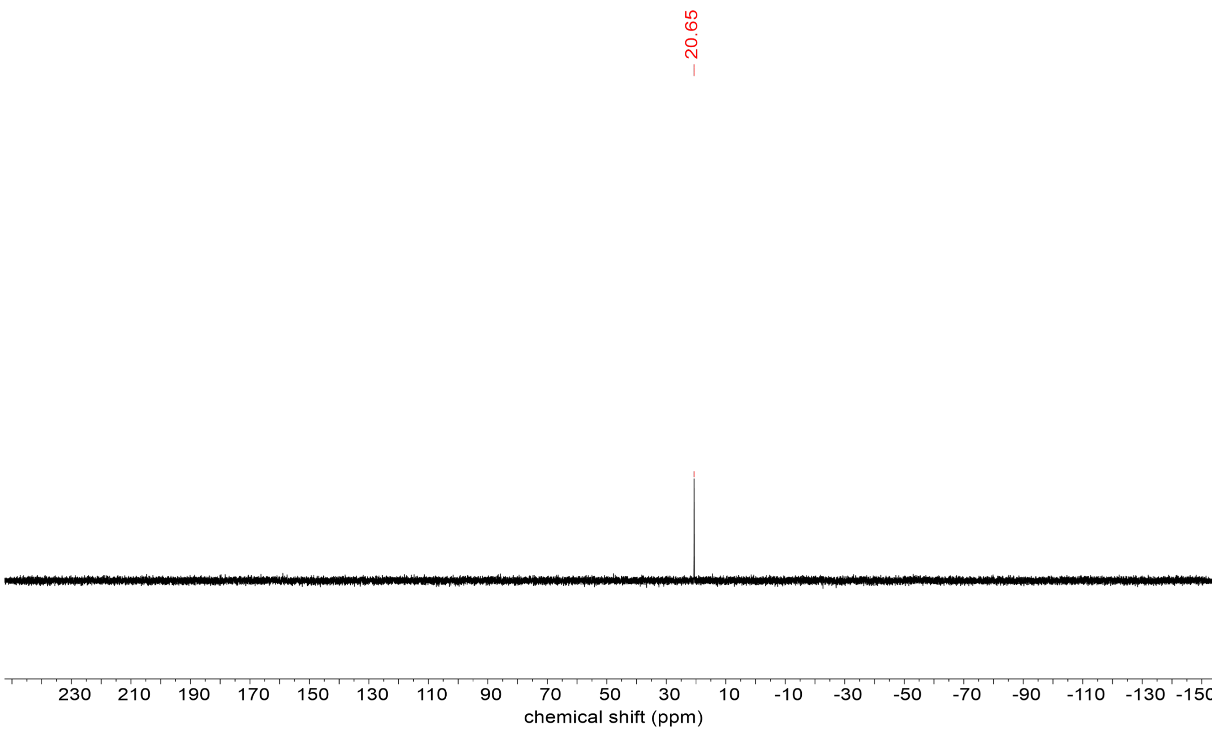

Figure S2. $^{31}P\{^{1}H\}$ NMR spectrum (162 MHz, CD_2Cl_2) of [^{t}BuHP$_1$pyrr][BPh_4].

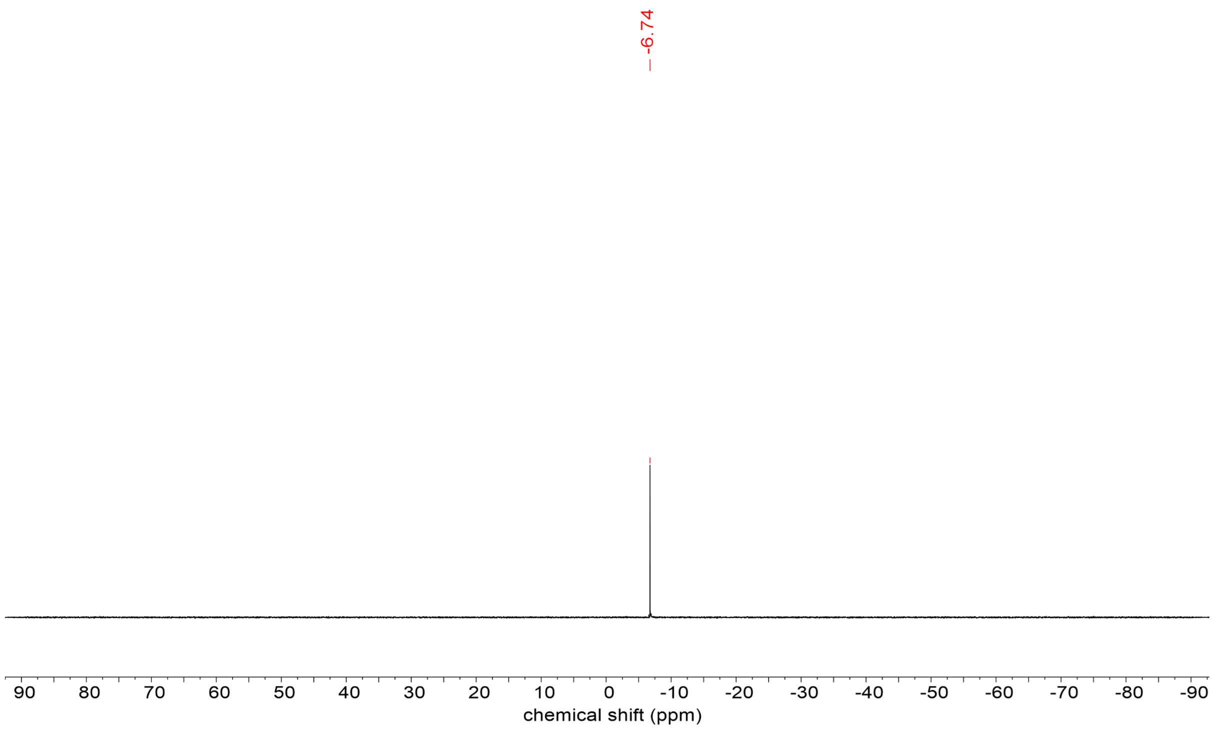

Figure S3. ^{11}B NMR spectrum (128 MHz, CD_2Cl_2) of [^{t}BuHP$_1$pyrr][BPh_4].

2. Cyclic Voltammetry & Open-Circuit Potential

Open-circuit potential (OCP) and cyclic voltammetry (CV) experiments were performed in a nitrogen-filled glovebox, unless otherwise noted, using a CHI 620E potentiostat (CH instruments Inc., Austin TX). The Pt wire (counter electrode) was flame-dried using a Bunsen burner. The glassy carbon electrode (working electrode) was polished with MicroPolish Powder (0.05 micron), rinsed with DI water, and then dried carefully with a Kimwipe prior to use. The Ag/$AgNO_3$ pseudoreference electrode was prepared in THF by stirring $AgNO_3$ (10 mM) in 100 mM electrolyte solution ([^{n}Bu_4N][PF_6]) overnight. An additional 20 mL of 100 mM electrolyte solution ([^{n}Bu_4N][PF_6]) was also prepared and stored over freshly activated 3 Å molecular sieves overnight. The $AgNO_3$ solution was filtered prior to adding to the reference electrode. The electrode was then rinsed and stored in the 20 mL 100 mM ([^{n}Bu_4N][PF_6]) electrolyte solution.

2.1. E_{OCP} (H^+/H_2) vs $Fc^{+/0}$

In a fume hood, a 20 mL electrolyte/ buffer stock solution of 100 mM [^{n}Bu_4N][PF_6], 50 mM lutidine, and 50 mM of [Hlut][BPh_4] in THF was prepared. [^{n}Bu_4N][PF_6], lutidine, and [Hlut][BPh_4] were measured in separate scintillation vials and dissolved in the electrolyte/ buffer solution.

The electrochemical cell was assembled by modifying previous reported procedures.[7] 5 mL of the buffer/electrolyte stock solution was added to the electrochemical cell containing two Pt wires (working electrode and counter electrode, as in a classic standard hydrogen electrode) and the Ag^+/Ag pseudoreference electrode. The Pt wires were flame-dried using a Bunsen burner before placing in the electrochemical cell. A gentle flow of H_2 was bubbled through the stirring electrochemical cell solution using a Schlenk line connected to an ultra-high purity H_2 tank. Figure S4 demonstrates the set up for determining the OCP of hydrogenation (H^+/H_2).

After 10 minutes of continuous H_2 sparging into the stirring solution, the OCP was measured in 1 s intervals for a total of 400 s. Once the OCP measurement was completed, the stirring was stopped, and ferrocene (Fc) was added to the electrochemical cell. A CV was taken by replacing the Pt wire, acting as a working electrode, with a glassy carbon electrode to reference

the OCPs accordingly. The experimental procedure was repeated for a total of three trials. The average OCP determined between the three trials was -0.469 V vs $Fc^{+/0}$. Figure S5 shows the representative E_{OCP} (H^+/H_2) vs $Fc^{+/0}$ data for Trial 2.

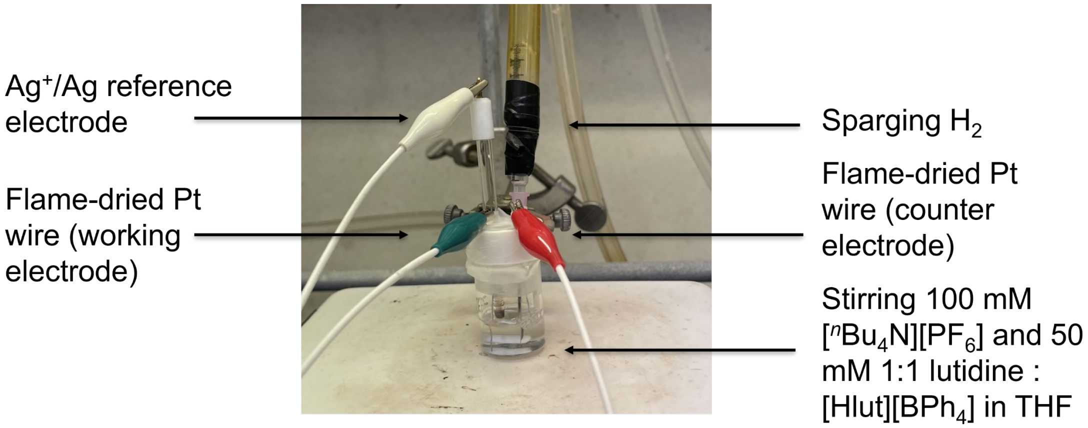

Figure S4. Experimental set up for OCP of hydrogenation (H^+/H_2).

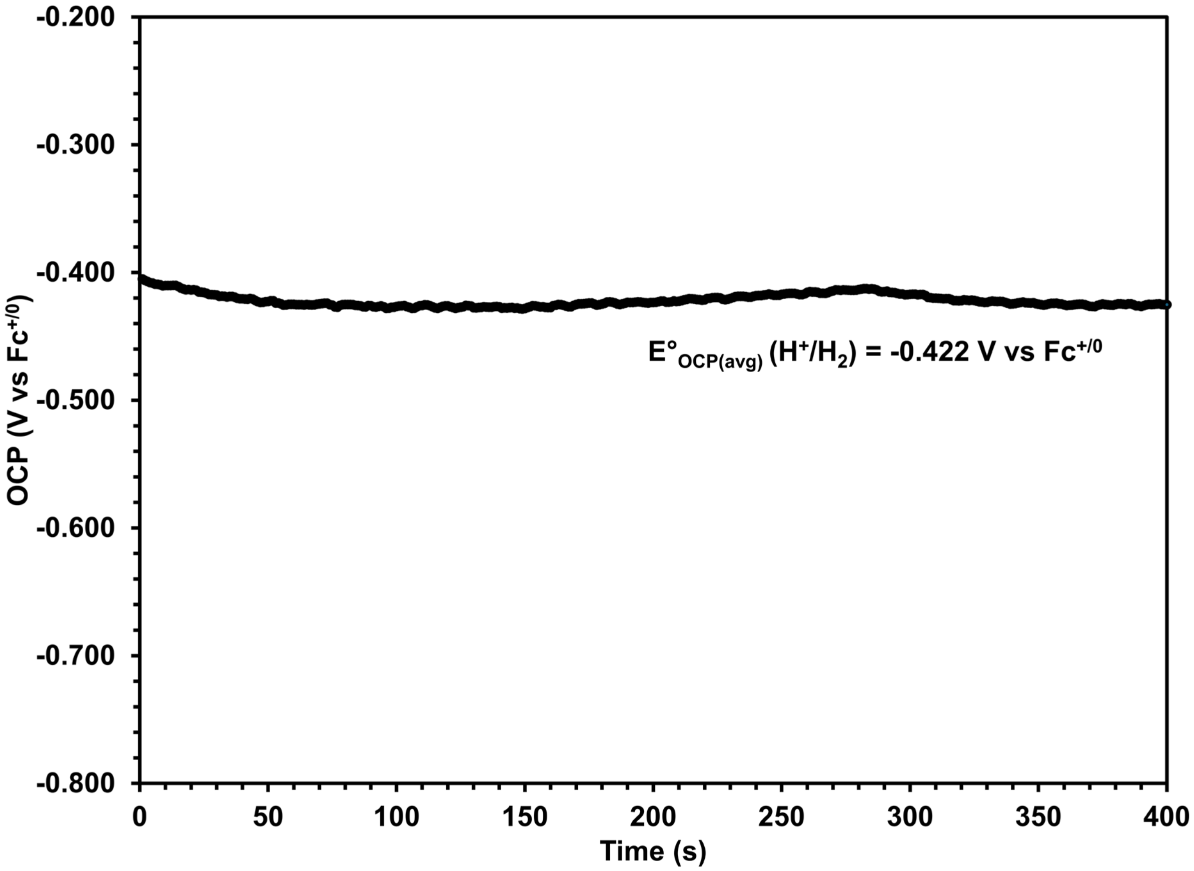

Figure S5. Trial 2: OCP of hydrogenation over time via continuous sparging of H_2 in a THF solution containing 100 mM [$^{n}Bu_4N$][PF_6], 50 mM lutidine, and 50 mM of [Hlut][BPh_4]. The OCP was measured every 1 s for 400 s.

2.2. E_{OCP} (O/OH) vs H_2

In a nitrogen-filled glovebox, OCP measurements between the hydroxide and oxo complexes (HO–Zr(MesNP^{i}Pr$_2$)$_3$CoCN^{t}Bu (**1**) and O≡Zr(MesNP^{i}Pr$_2$)$_3$CoCN^{t}Bu (**2**), respectively) were collected following published procedures.[7] An electrolyte/buffer stock solution of 100 mM [^{n}Bu$_4$N][PF$_6$], 50 mM lutidine, and 50 mM [Hlut][BPh$_4$] in THF was prepared using a 100 mL volumetric flask. [^{n}Bu$_4$N][PF$_6$], lutidine, and [Hlut][BPh$_4$] were measured in separate scintillation vials, dissolved in the electrolyte/buffer solution, and transferred to the volumetric flask. The electrolyte/ buffer stock solution was stored over 3 Å molecular sieves prior to use. Two separate stock solutions of complexes **1** and **2** were prepared using 5 mL volumetric flasks. The previously mentioned electrolyte/ buffer solution containing 100 mM [^{n}Bu$_4$N][PF$_6$], 50 mM lutidine, 50 mM [Hlut][BPh$_4$] in THF was used to dilute to the mark. Solutions of desired **1** and **2** concentrations were prepared using the stock solutions described above. A glass syringe was used to deliver the appropriate volumes of the **1** and **2** stock solutions into a 5 mL volumetric flask. The same electrolyte/buffer stock solution was used to dilute to the mark.

In an electrochemical cell containing the glassy carbon electrode (working electrode), the Ag^+/Ag pseudoreference electrode, and the Pt wire (counter electrode), 5 mL of the hydroxide (**1**)/ oxo (**2**) solution was added. OCP measurements were collected using five ratios of **1**:**2** (approximately 1.0 mM : 1.5 mM, 1.25 mM : 1.5 mM, 1.5 mM : 1.5 mM, 1.75 mM : 1.5 mM, 2.0 mM : 1.5 mM, respectively). While stirring, OCP measurements were taken in 1 s intervals for 400 s. After each OCP run, the stirring was stopped, and Fc was added to the electrochemical cell to collect a CV to reference the OH/O OCP vs $Fc^{+/0}$. The OCP measurement at each **1**:**2** ratio was repeated to confirm reproducibility.

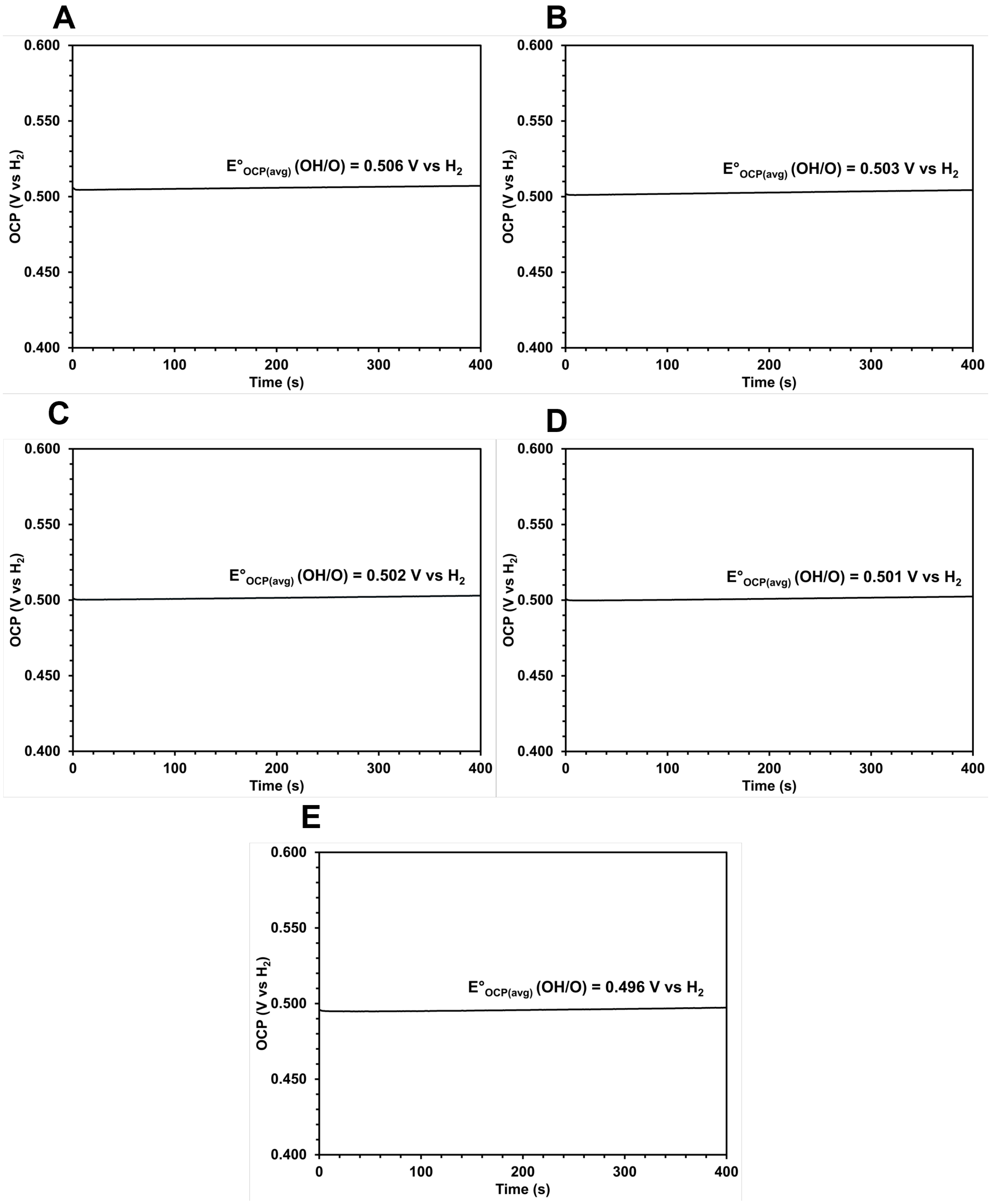

Figure S6. Trial 1: OCP (V vs H_2) over time of THF solutions containing 100 mM [^{n}Bu$_4$N][PF$_6$], 50 mM lutidine, 50 mM of [Hlut][BPh$_4$], and varying ratios of **1** and **2**. The OCP was measured every 1 s for 400 s. Each ratio is represented as follows: A) 0.95:1.42, B) 1.19:1.42, C) 1.43:1.42, D) 1.67:1.42, and E) 1.90:1.42.

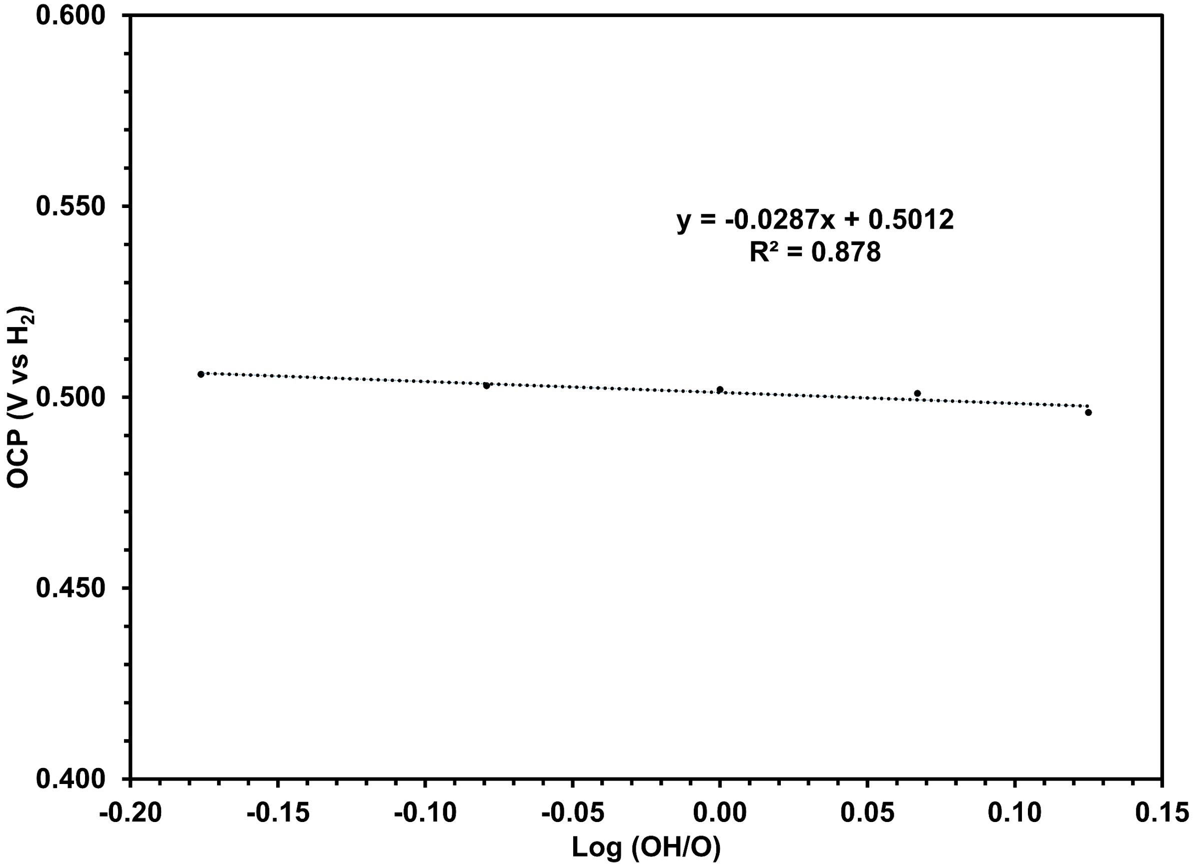

Figure S7. Trial 1: OCP (V vs H_2) vs. the log of varying ratios of complexes **1** and **2**. OCPs were determined by using the $E°_{OCP(avg)}$ (OH/O) values at each given ratio from Figure S6.

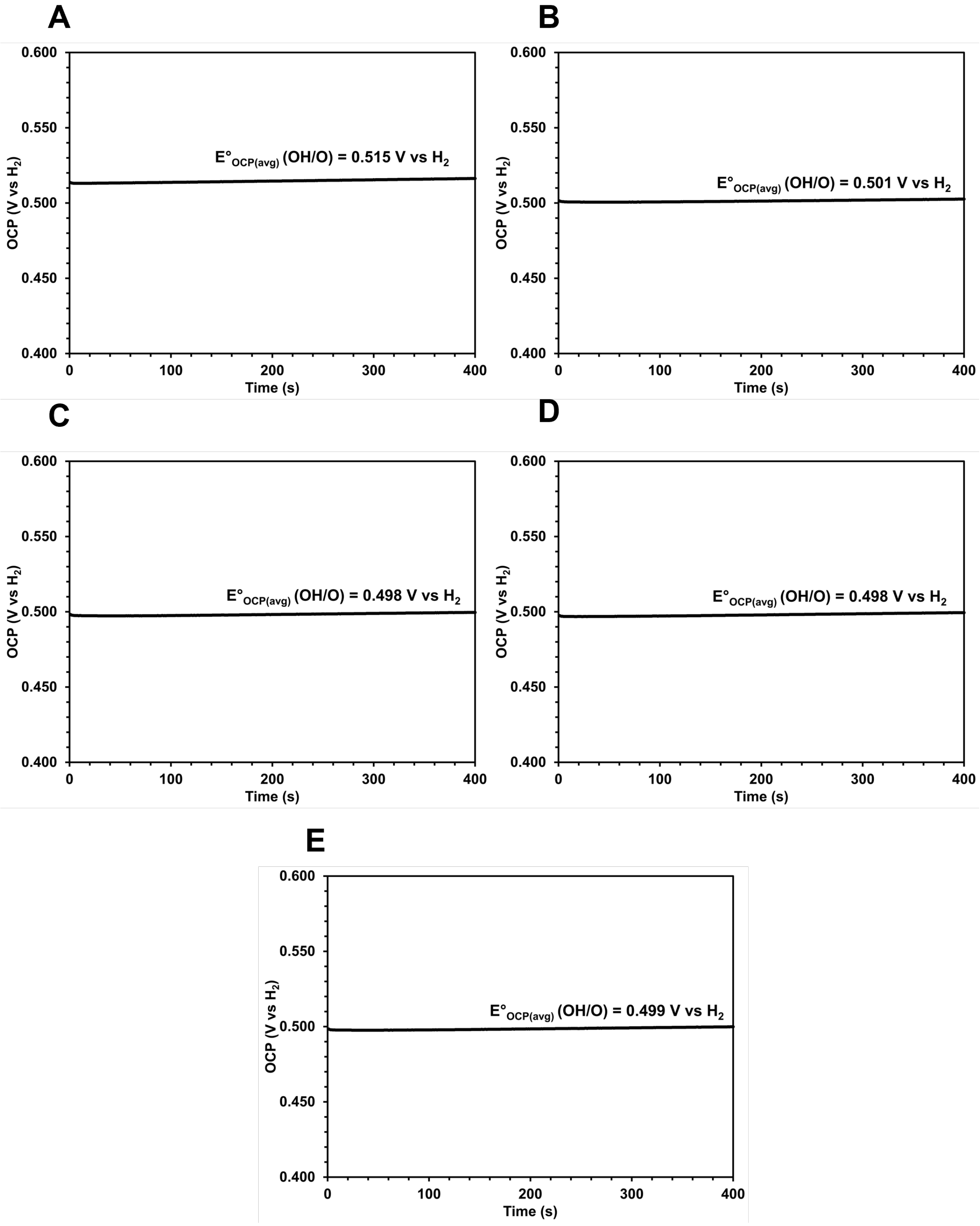

Figure S8. Trial 2: OCP (V vs H_2) over time of THF solutions containing 100 mM [^{n}Bu$_4$N][PF$_6$], 50 mM lutidine, 50 mM of [Hlut][BPh$_4$], and varying ratios of **1** and **2**. The OCP was measured every 1 s for 400 s. Each ratio is represented as follows: A) 0.95:1.42, B) 1.19:1.42, C) 1.43:1.42, D) 1.67:1.42, and E) 1.90:1.42.

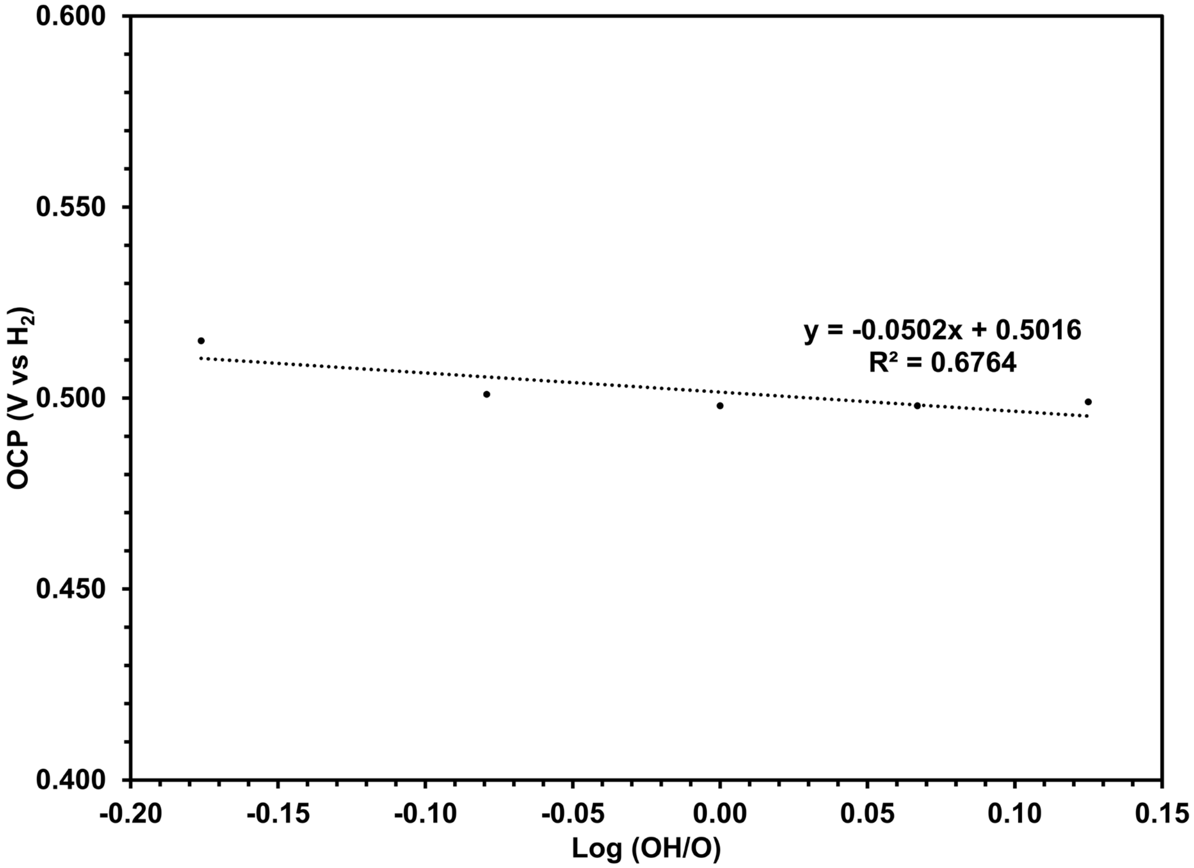

Figure S9. Trial 2: OCP (V vs H_2) vs. the log of varying ratios of complexes **1** and **2**. OCPs were determined by using the $E°_{OCP(avg)}$ (OH/O) values at each given ratio from Figure S8.

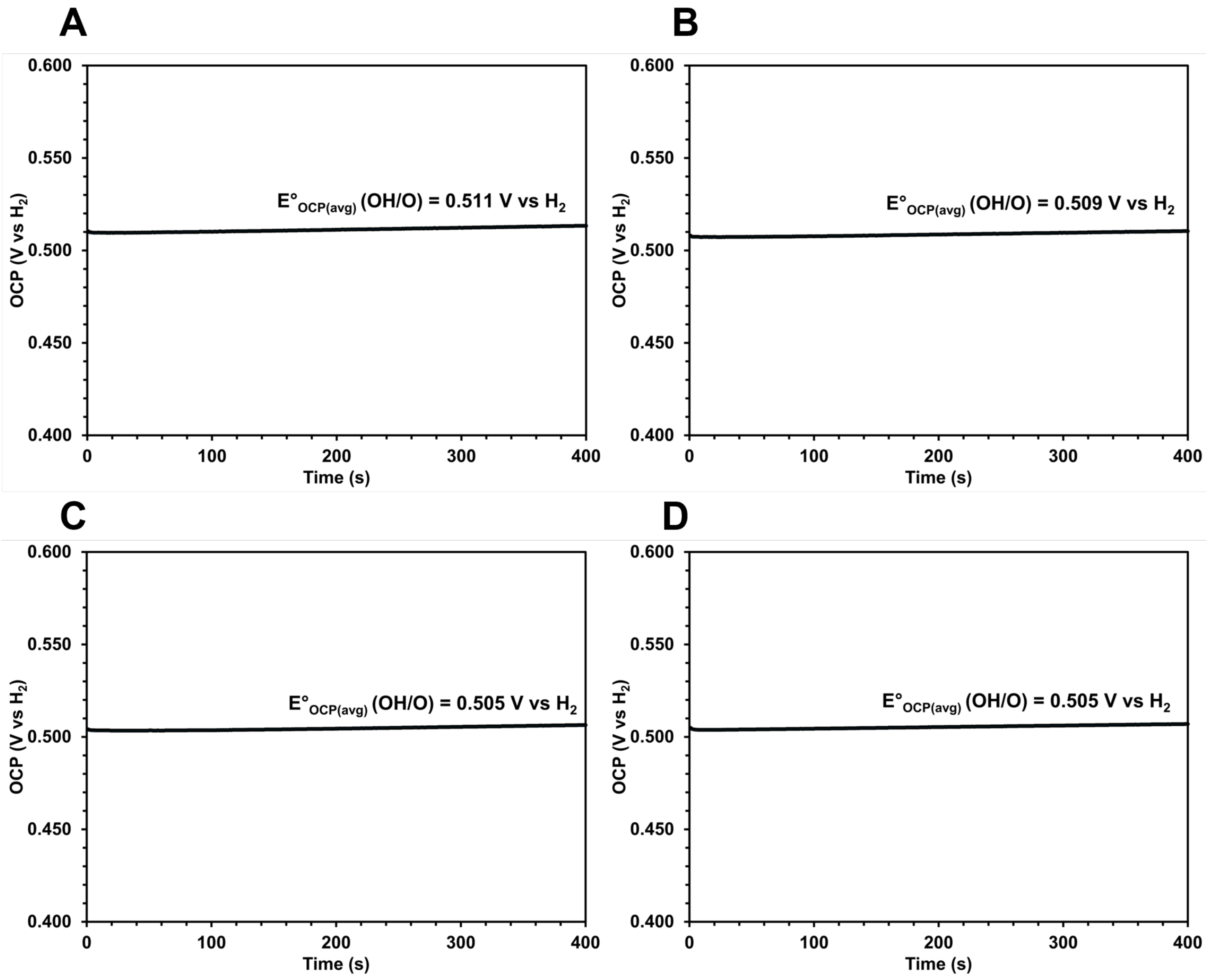

Figure S10. Trial 3: OCP (V vs H_2) over time of THF solutions containing 100 mM [^{n}Bu$_4$N][PF$_6$], 50 mM lutidine, 50 mM of [Hlut][BPh$_4$], and varying ratios of **1** and **2**. The OCP was measured every 1 s for 400 s. Each ratio is represented as follows: A) 0.95:1.42, B) 1.19:1.42, C) 1.43:1.42, and D) 1.67:1.42.

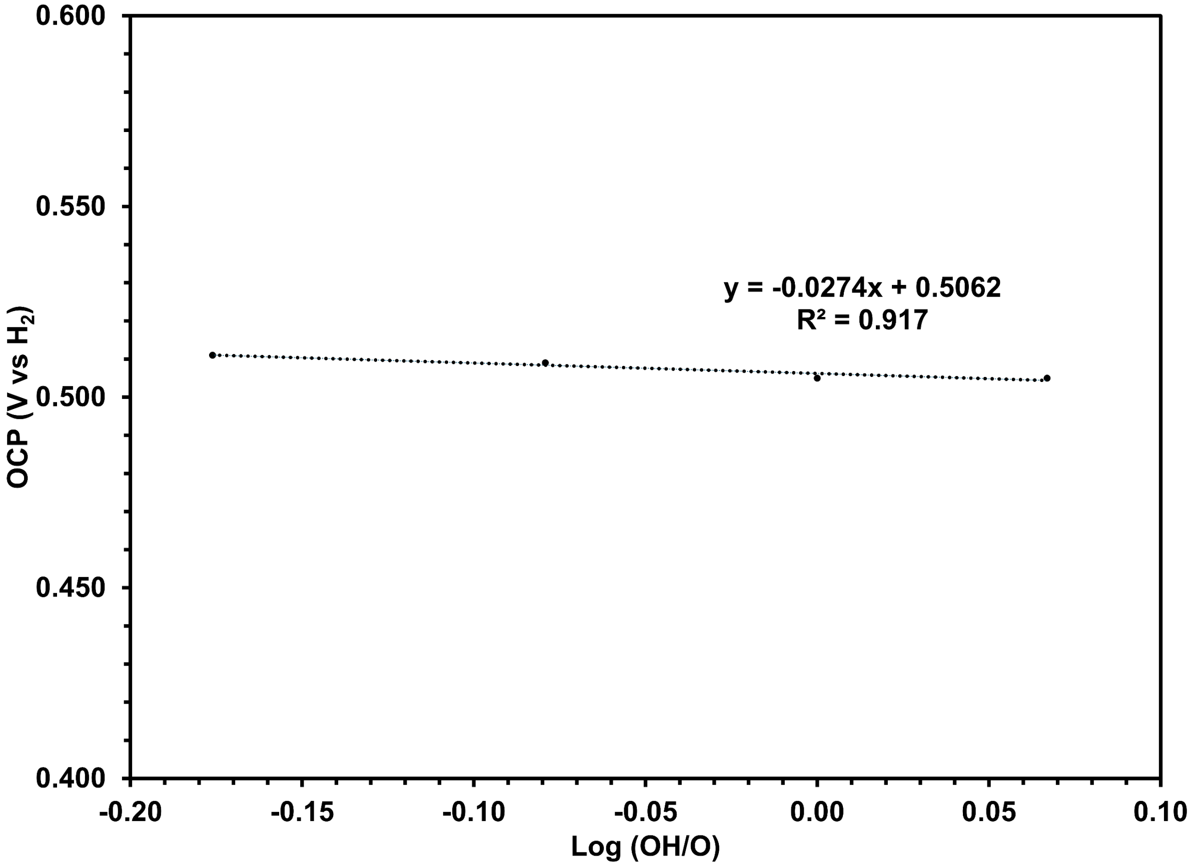

Figure S11. Trial 3: OCP (V vs H_2) vs. the log of varying ratios of complexes **1** and **2**. OCPs were determined by using the $E°_{OCP(avg)}$ (OH/O) values at each given ratio from Figure S10.

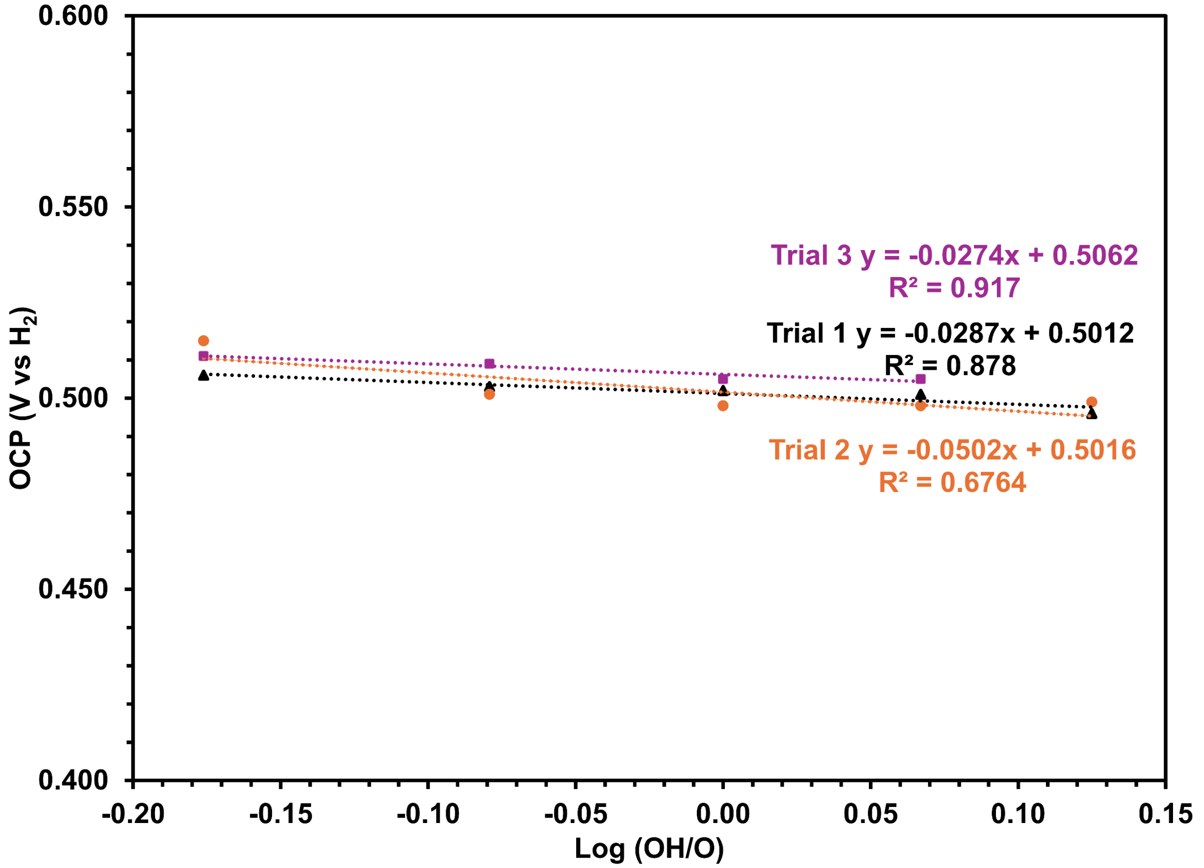

Figure S12. Summary of the OCP (V vs H_2) over the log of varying ratios of complexes **1** and **2**. OCPs were determined by using the $E°_{OCP(avg)}$ (OH/O) values at each given ratio from Figure S6, Figure S8, and Figure S10.

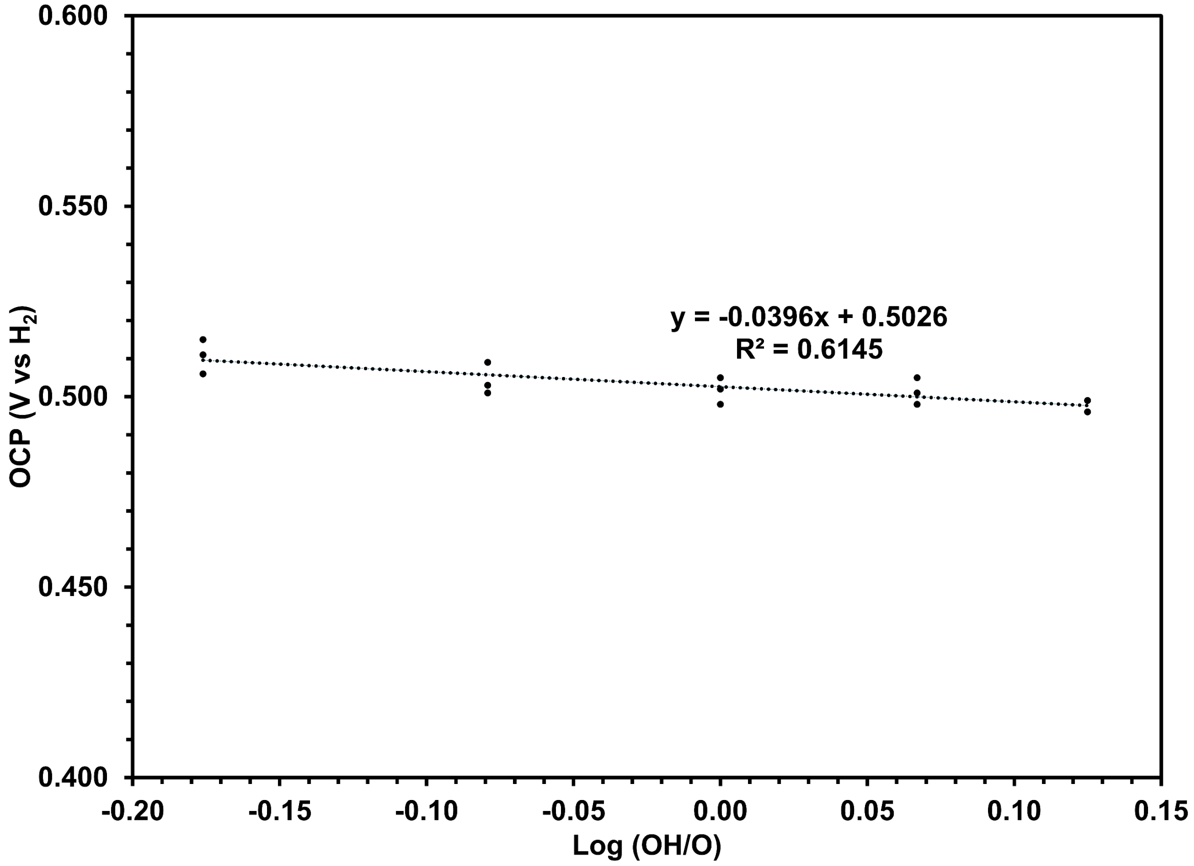

Figure S13. Data points for all three trials combined into a single plot of the OCP (V vs H_2) vs the log of varying ratios of complexes **1** and **2**. OCPs were determined by using the $E°_{OCP(avg)}$ (OH/O) values at each given ratio from Figure S6, Figure S8, and Figure S10.

3. Direct conversion of OCP (V vs H_2) to $BDFE_{O\text{-}H}$

$$X + nHA + ne^- \xrightleftharpoons{E^{\circ\prime}_{OCP}\ (V\ vs\ Fc^+/Fc)} XH_n + nA^-$$

$$(^n/_2)H_{2(g)} + nA^- \xrightleftharpoons{-E^{\circ\prime}\ (H^+/H_2,\ V\ vs\ Fc^+/Fc)} nHA + ne^-$$

$$X + (^n/_2)H_{2(g)} \xrightleftharpoons{E^{\circ\prime}\ (X/XH_n,\ V\ vs\ H_2)} XH_n$$

Scheme S1. Direct conversion of OCP (V vs H_2) to $BDFE_{X\text{-}H}$. Reprinted (adapted) with permission from Wise, C. F.; Agarwal, R. G.; Mayer, J. M.; *J. Am. Chem. Soc.*, **2020**, *142*, 10681-10691. Copyright 2020 American Chemical Society.

$$\mathbf{BDFE(XH) = 23.06E^\circ(X/XH\ V\ vs\ H_2) + \Delta G^\circ(\tfrac{1}{2}H_{2(g)})/H^{\cdot}_{1M}} \quad \textbf{(eq. S1)}$$

By referencing the X/XH OCP to the OCP of the H^+/H_2 solution, a direct route (Scheme S1) can be used to calculate the OCP of X/XH vs H^+/H_2 (E° (V vs H_2)).[7] The $BDFE_{X\text{-}H}$ can then be determined by substituting the E° (V vs H_2) in equation S1,[7] since the value for $\Delta G^\circ(\frac{1}{2}H_2\ (g)/H^{\cdot}_{1M})$ is known to be 52.0 kcal/mol in THF.[7] By determining the $BDFE_{X\text{-}H}$ with OCP measurements, the pK_a of the complexes can then be estimated using the Bordwell equation (eq. S2),[8,9] in which the solvent-specific constant ($C_{g,sol}$) is 59.9 kcal/mol in THF[10], as demonstrated below with sample calculations. Redox potentials (included in Scheme 3 in main text) for complexes **1** and **2** were previously determined via CVs.[1,2]

3.1. $BDFE_{O\text{-}H}$ sample calculation

$$\mathbf{BDFE(OH) = 23.06(0.503) + \Delta G^\circ(52)}$$

$$\mathbf{BDFE(OH) = 63.6\ kcal/mol}$$

$$\mathbf{BDFE(OH) = 64\ \pm 1\ kcal/mol}$$

3.2. pK_a sample calculation

$$\mathbf{BDFEsol(X-H) = 1.37pK_a + 23.06E^\circ + C_{G,sol}}\ \textbf{(eq. S2)}$$

$$\mathbf{pK_a = \frac{BDFE(XH) - C_{G,sol} - 23.06E^\circ(X^{0/-})}{1.37}}\ \textbf{(eq. S2)}$$

$$\mathbf{pK_a = \frac{63.6 - 59.9 - 23.06(-1.71)}{1.37} = 31.5}$$

$$\mathbf{pK_a = \frac{63.6 - 59.9 - 23.06(-1.13)}{1.37} = 21.7}$$

3.3. Uncertainty in $BDFE_{O-H}$

The uncertainty in $BDFE_{O-H}$ was estimated using the experimentally observed deviations in E_{OCP} (H^+/H_2) vs $Fc^{+/0}$ and E_{OCP} (O/OH) vs $Fc^{+/0}$. A standard deviation of 40.5 mV was observed for E_{OCP} (H^+/H_2) vs $Fc^{+/0}$ measurements. A standard deviation of 2.8 mV was observed for E_{OCP} (O/OH) vs $Fc^{+/0}$ measurements. Taking these deviations as additive, a total deviation of 43.3 mV corresponds to a BDFE uncertainty of 1.0 kcal/mol when multiplied by the 23.06 conversion factor. The estimated uncertainty is similar to previously reported BDFE uncertainty values of 1.0[7] and 1.3[11] kcal/mol, which also include temperature differences and electrode drift over multiple days. The OCP measurements reported herein were collected on the same day using the same electrodes to minimize temperature differences and electrode drift.

4. BDFE$_{O–H}$ test reactions

4.1. HO–Zr(MesNP^{i}Pr$_2$)$_3$CoCN^{t}Bu (1) with 2,4,6-tri-tert-butylphenoxyl radical

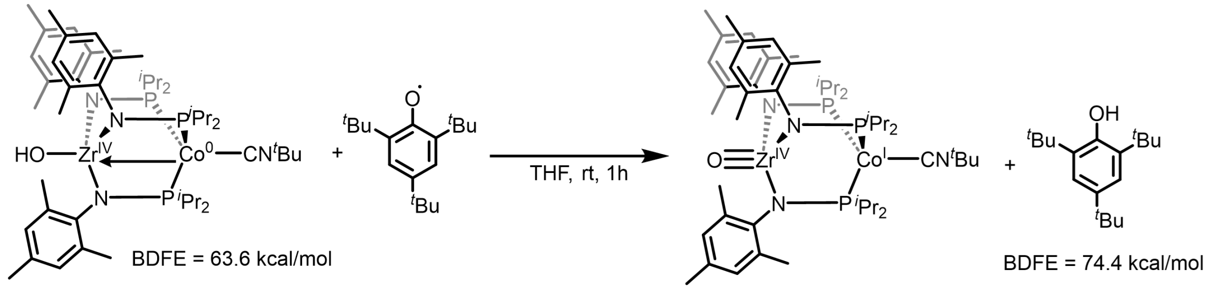

The 2,4,6-tri-tert-butylphenoxyl radical (5.9 mg, 0.023 mmol) was dissolved in THF (~2 mL) and added to a stirring solution of complex **1** (22.7 mg, 0.0227 mmol) in THF (~2 mL). A bright green suspension was observed. The reaction mixture was allowed to stir at room temperature for 1 h. The THF was removed under vacuum prior to ^{1}H NMR analysis.

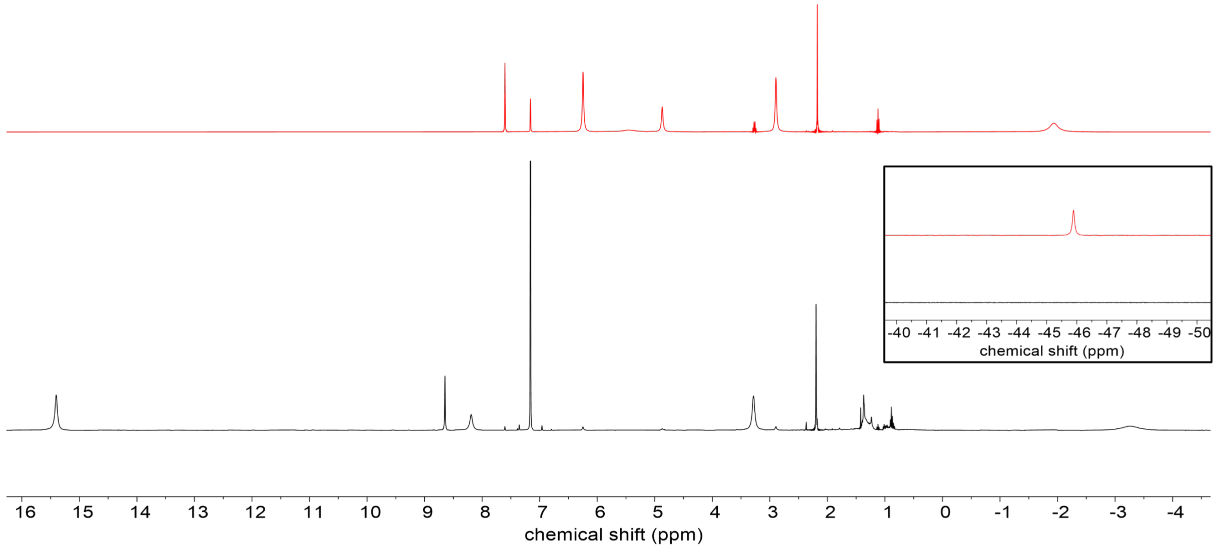

Figure S14. ^{1}H NMR spectra (400 MHz, C_6D_6) of hydroxide starting material **1** (top, red) and the crude reaction between **1** and the 2,4,6-tri-tert-butylphenoxyl radical after 1 h of stirring (bottom, black). Inset shows the region containing the hydroxide proton in **1**.

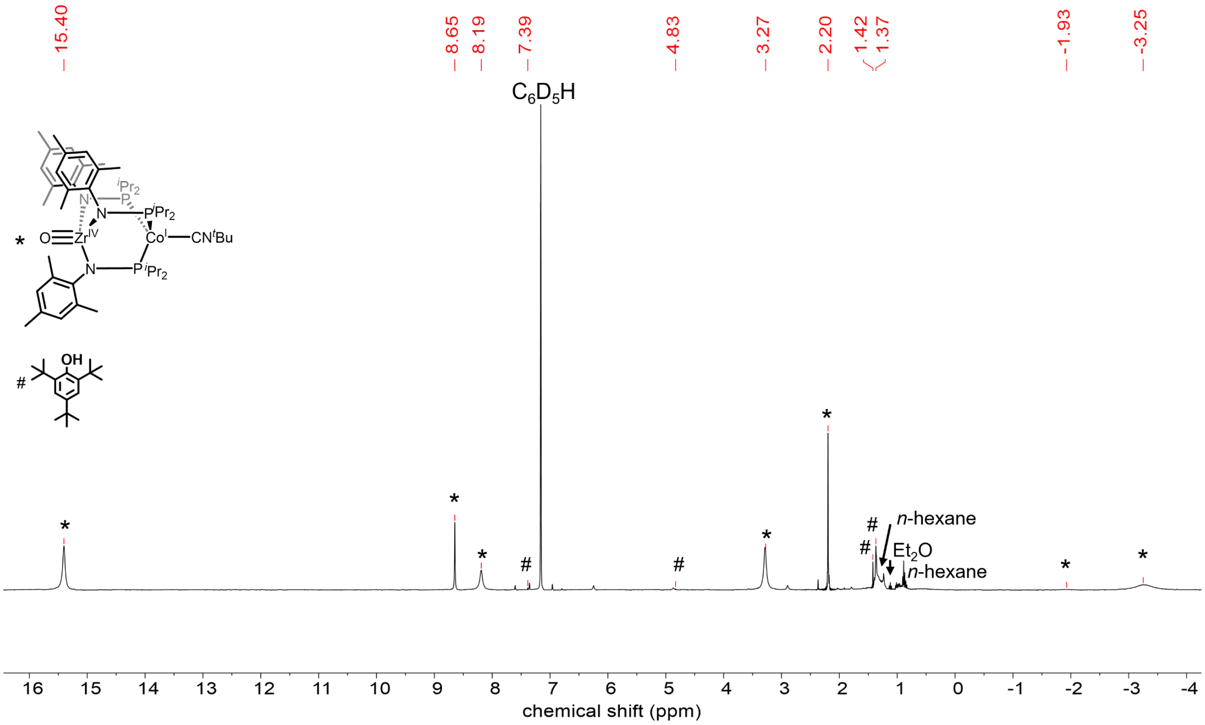

Figure S15. ^{1}H NMR spectrum (400 MHz, C_6D_6) of the crude reaction between **1** and the 2,4,6-tri-tert-butylphenoxyl radical after 1 h of stirring.

4.2. O≡Zr(MesNP^{i}Pr$_2$)$_3$CoCN^{t}Bu (2) with 9,10-dihydroanthracene

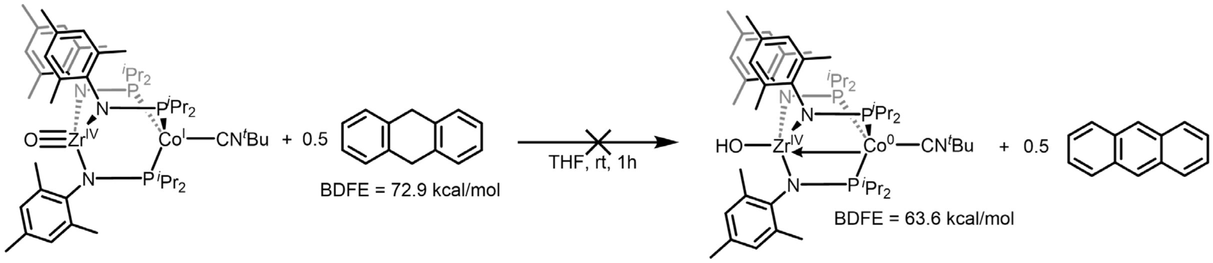

9,10-dihydroanthracene (2.2 mg, 0.012 mmol) was dissolved in THF (~2 mL) and added to a stirring solution of complex **2** (10.8 mg, 0.0108 mmol) in THF (~2 mL). There was no color change observed; the reaction mixture remained bright green. The reaction mixture was allowed to stir at room temperature for 1 h. The THF was removed under vacuum prior to ^{1}H NMR analysis.

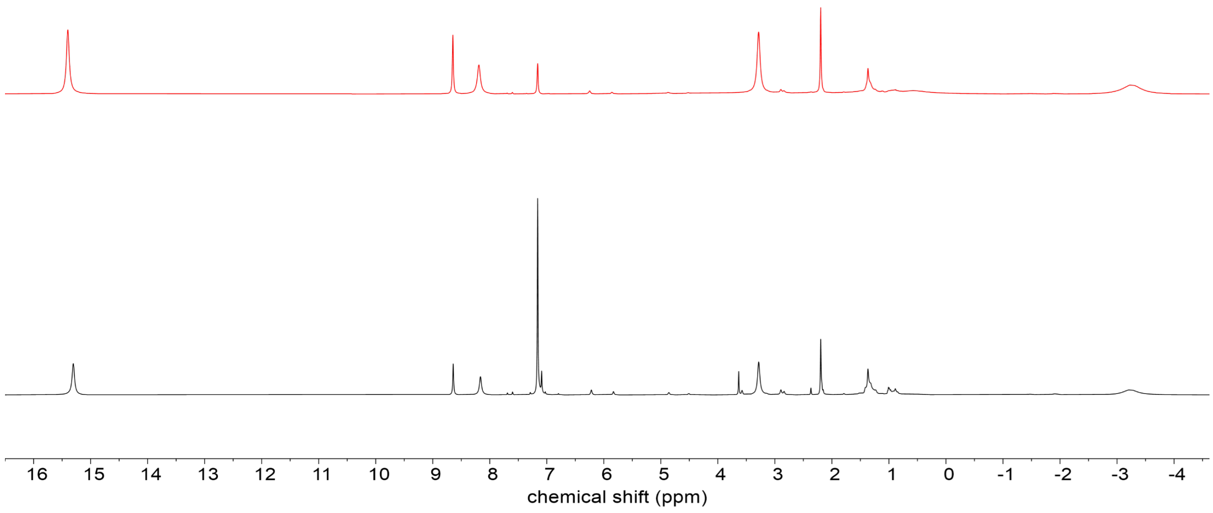

Figure S16. ^{1}H NMR spectra (400 MHz, C_6D_6) of oxo starting material **2** (top, red) and the crude reaction between **2** and 9,10-dihydroanthracene after 1 h of stirring (bottom, black).

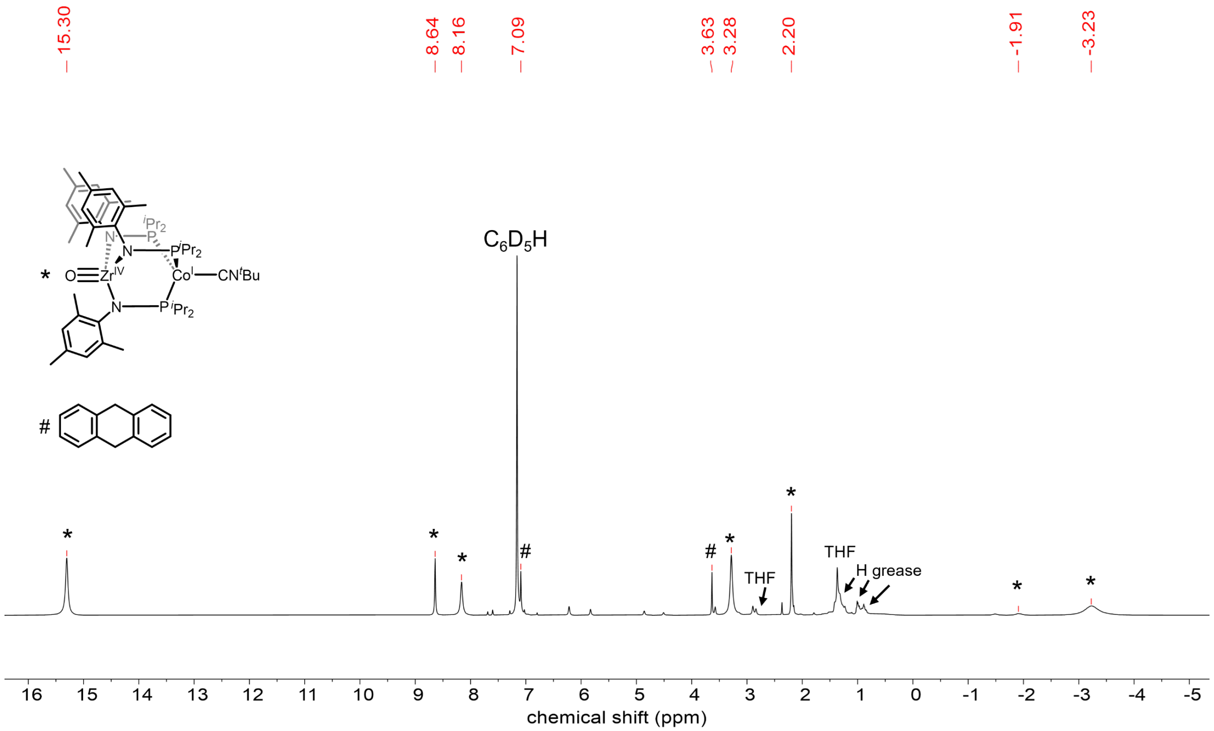

Figure S17. ^{1}H NMR spectrum (400 MHz, C_6D_6) of the crude reaction between **2** and 9,10-dihydroanthracene after 1 h of stirring.

4.3. HO–Zr(MesNP^{i}Pr$_2$)$_3$CoCN^{t}Bu (1) with *p*-benzoquinone

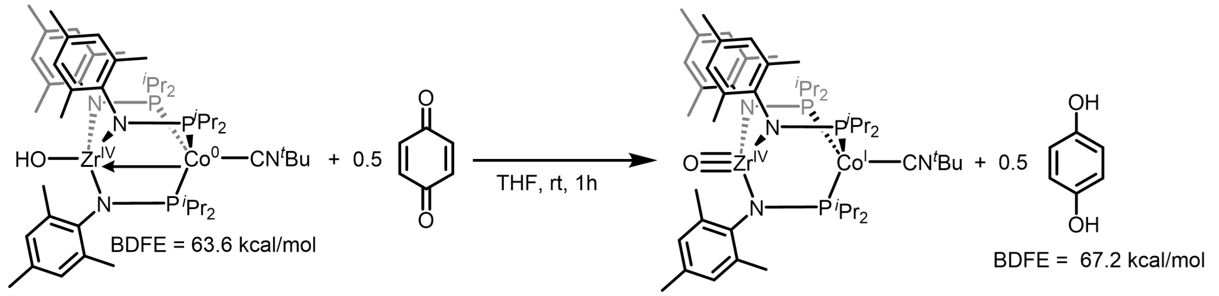

p-Benzoquinone (1.5 mg, 0.014 mmol) was dissolved in THF (~2 mL) and added to a stirring solution of complex **1** (10.7 mg, 0.0107 mmol) in THF (~2 mL). A color change from yellow to green was observed with a bright green solid precipitating out of solution. The reaction mixture was allowed to stir at room temperature for 1 h. The THF was removed under vacuum prior to ^{1}H NMR analysis.

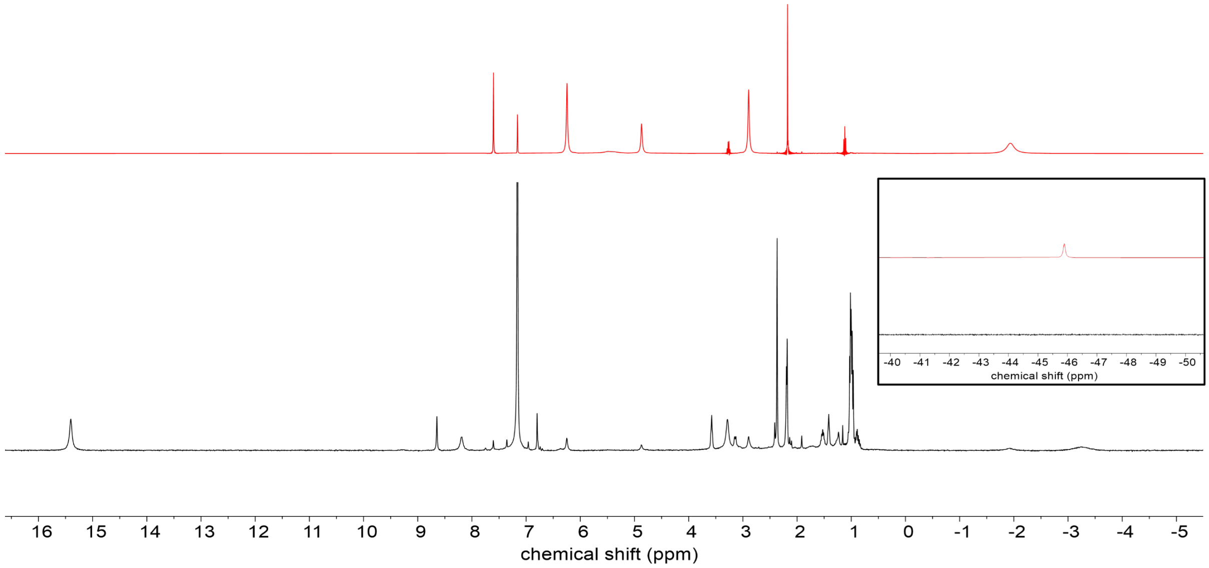

Figure S18. ^{1}H NMR spectra (400 MHz, C_6D_6) of hydroxide starting material **1** (top, red) and the crude reaction between **1** and *p*-benzoquinone after 1 h of stirring (bottom, black). Inset shows the region containing the hydroxide proton of **1**.

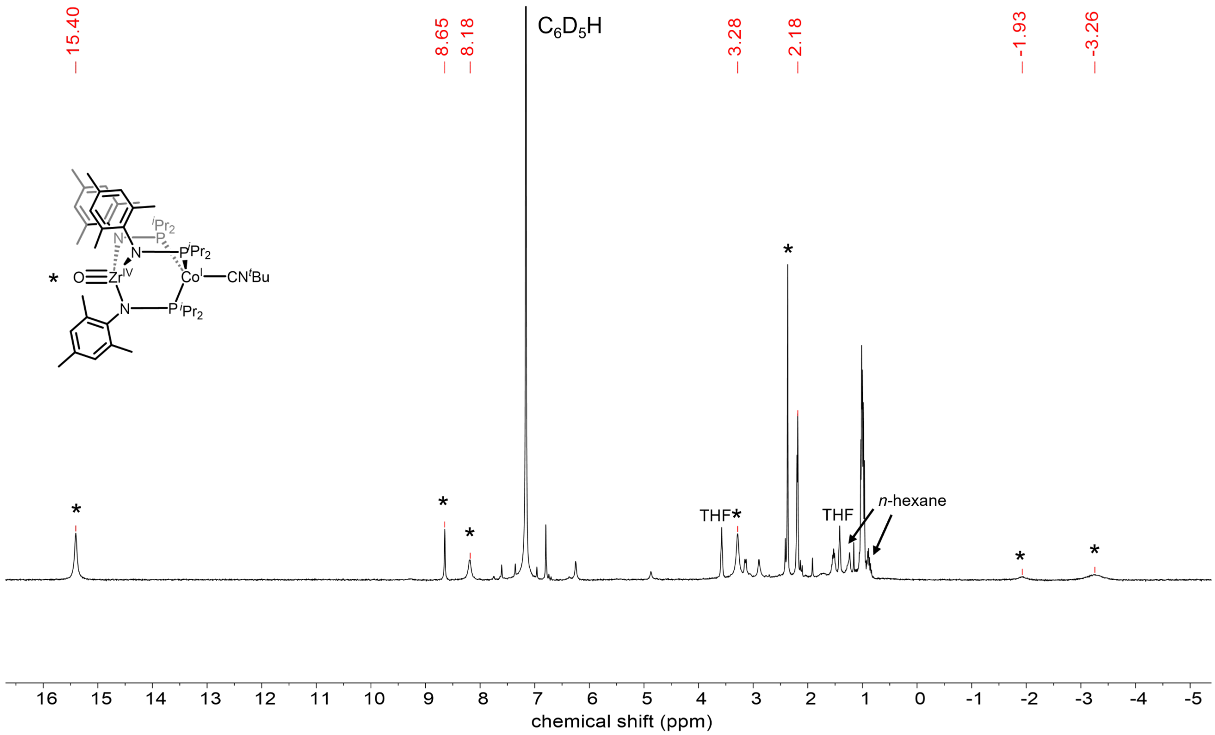

Figure S19. ^{1}H NMR spectrum (400 MHz, C_6D_6) of the crude reaction between **1** and *p*-benzoquinone after 1 h of stirring.

4.4. HO–Zr(MesNP^{i}Pr$_2$)$_3$CoCN^{t}Bu (1) with 1,8-dichloroanthraquinone

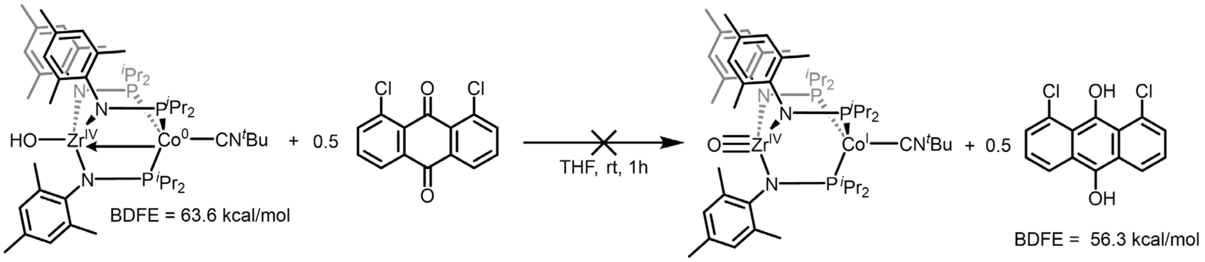

Complex **1** (10.0 mg, 0.00999 mmol) was dissolved in THF (~2 mL) and added to 1,8-dichloroanthraquinone (3.0 mg, 0.0108 mmol) with stirring. Within the first few minutes of the reaction, there was no color change observed, the solution remained yellow. After 20 minutes of stirring, the solution became a deep red/purple color. The reaction mixture was allowed to stir at room temperature for 1 h. The THF was removed under vacuum prior to ^{1}H NMR analysis. Since the ^{1}H NMR spectrum of the reaction mixture indicated that no reaction had occurred, the

red/purple color is posited to result from hydrogen bonding and formation of a weak donor/acceptor complex.

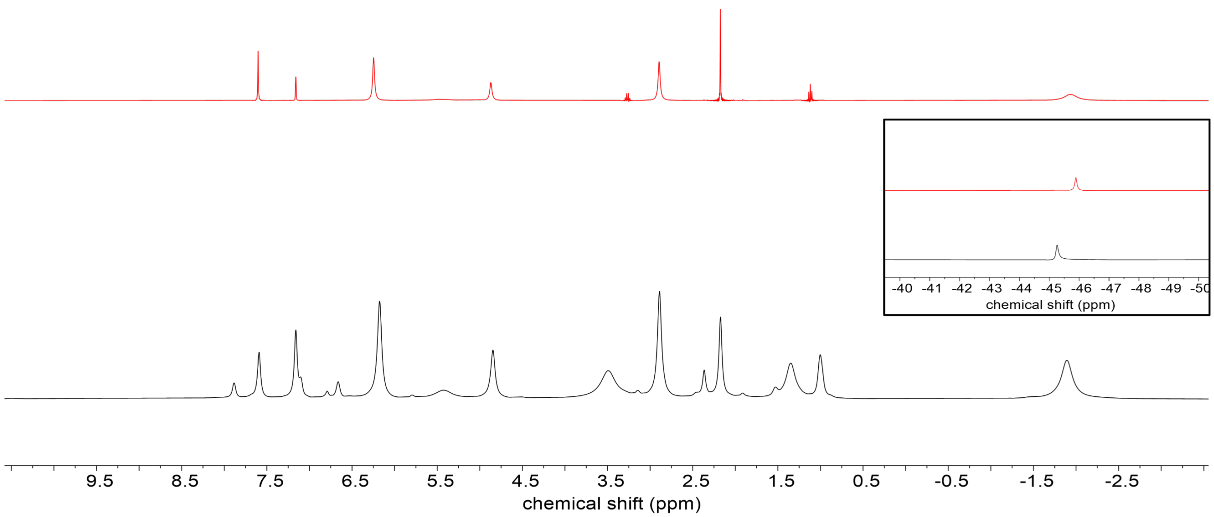

Figure S20. ${}^{1}H$ NMR spectra (600 MHz, C_6D_6) of hydroxide starting material **1** (top, red) and the crude reaction between **1** and 1,8-dichloroanthraquinone after 1 h of stirring (bottom, black). Inset shows the region containing the hydroxide proton of **1**.

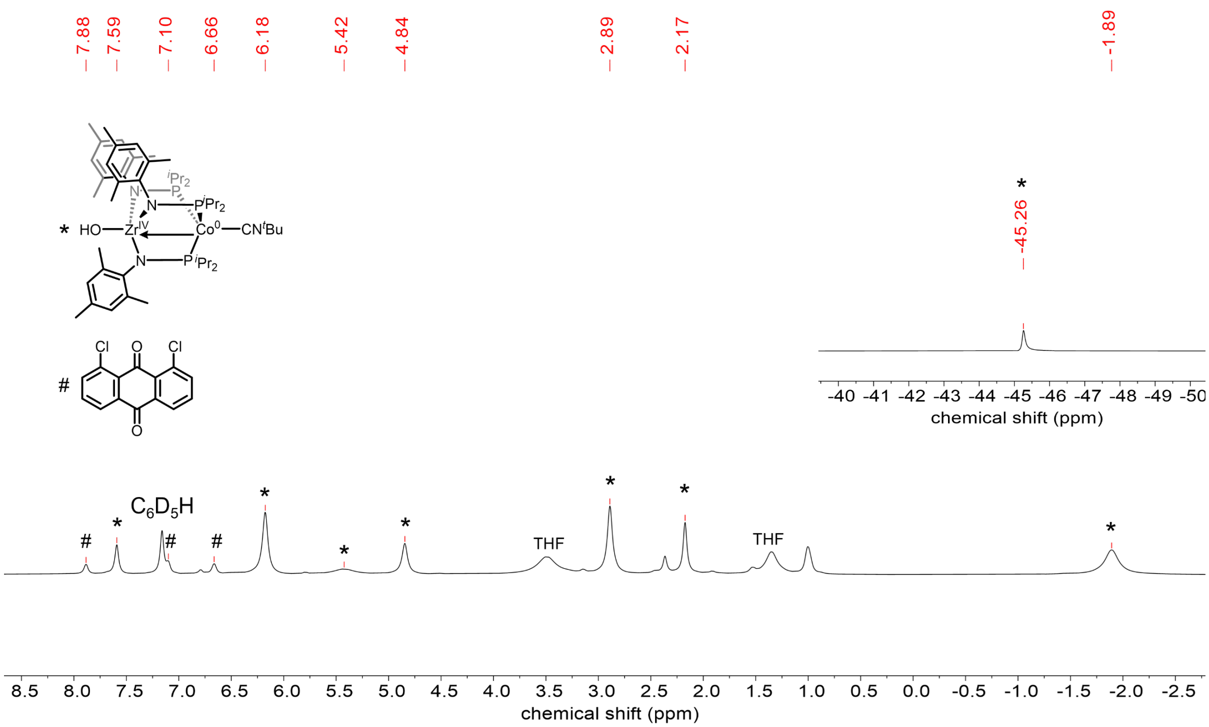

Figure S21. ^{1}H NMR spectrum (600 MHz, C_6D_6) of the crude reaction between **1** and 1,8-dichloroanthraquinone after 1 h of stirring.

5. pK_a test reactions

pK_a HO–Zr(MesNP^{i}Pr$_2$)$_3$CoCN^{t}Bu (1) = 31.5

5.1. [(μ-Na)OZr(MesNP^{i}Pr$_2$)$_3$CoCN^{t}Bu]$_2$ (2$^-$) with 4-methylpyridine

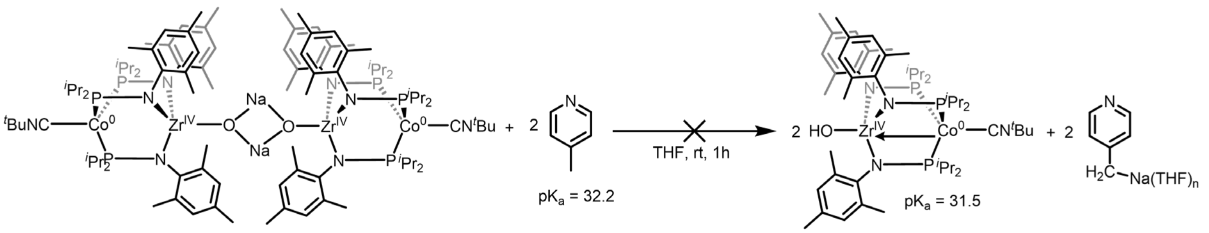

4-methylpyridine (1.1 mg, 0.012 mmol) was added to a stirring solution of **2**$^-$ (11.7 mg, 0.00572 mmol) in THF (~2 mL). There was no color change observed during the reaction; the solution remained yellow-orange. The reaction mixture was allowed to stir at room temperature for 1 h. The THF was removed under vacuum prior to ^{1}H NMR analysis.

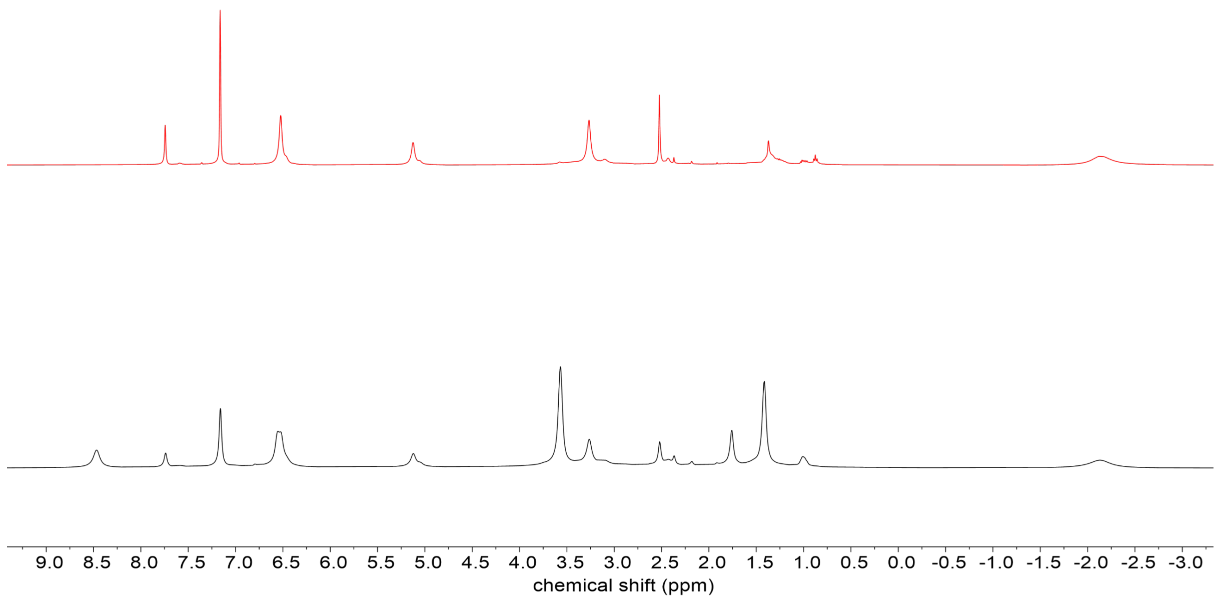

Figure S22. ^{1}H NMR spectra (400 MHz, C_6D_6) of **2**$^-$ (top, red) and the crude reaction between **2**$^-$ and 4-methylpyridine after 1 h of stirring (bottom, black).

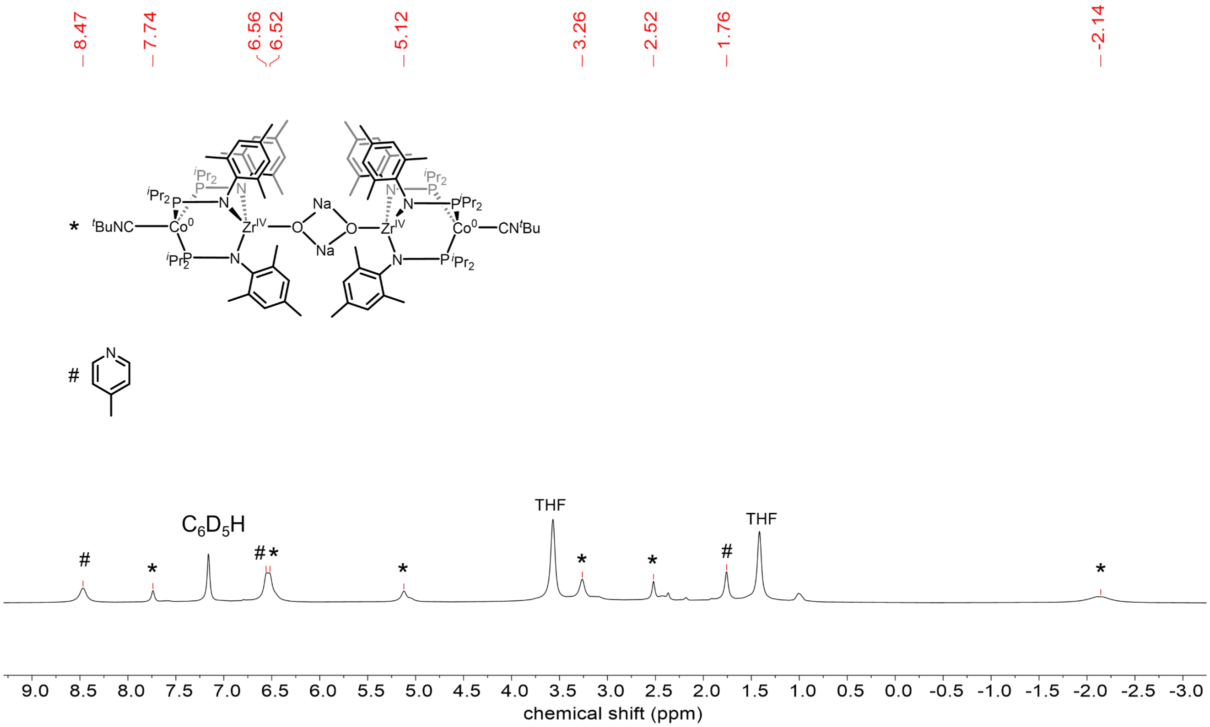

Figure S23. ^{1}H NMR spectrum (400 MHz, C_6D_6) of the crude reaction between **2**$^-$ and 4-methylpyridine after 1 h of stirring.

5.2. [(*μ*-Na)OZr(MesNP^{i}Pr$_2$)$_3$CoCN^{t}Bu]$_2$ (2$^-$) with [^{t}BuHP$_1$pyrr][BPh$_4$]

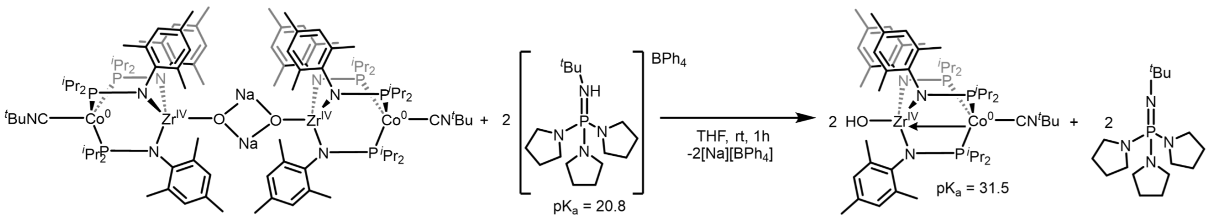

[^{t}BuHP$_1$pyrr][BPh$_4$] (3.3 mg, 0.0052 mmol) was dissolved in THF (~2 mL) and added to **2**$^-$ (4.3 mg, 0.0021 mmol) with stirring. A color change from orange to yellow was observed. The reaction mixture was allowed to stir at room temperature for 1 hour. The THF was removed under vacuum prior to ^{1}H NMR analysis.

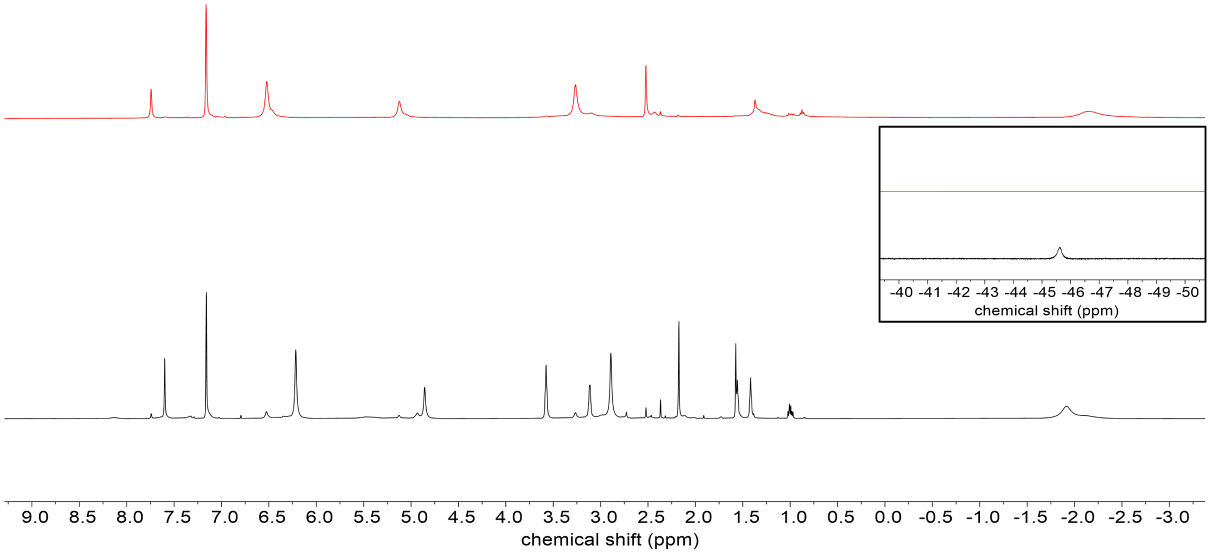

Figure S24. ^{1}H NMR spectra (600 MHz, C_6D_6) of **2**$^-$ (top, red) and the crude reaction between **2**$^-$ and [^{t}BuHP$_1$pyrr][BPh_4] after 1 h of stirring (bottom, black). Inset shows the region containing the hydroxide proton of **1**.

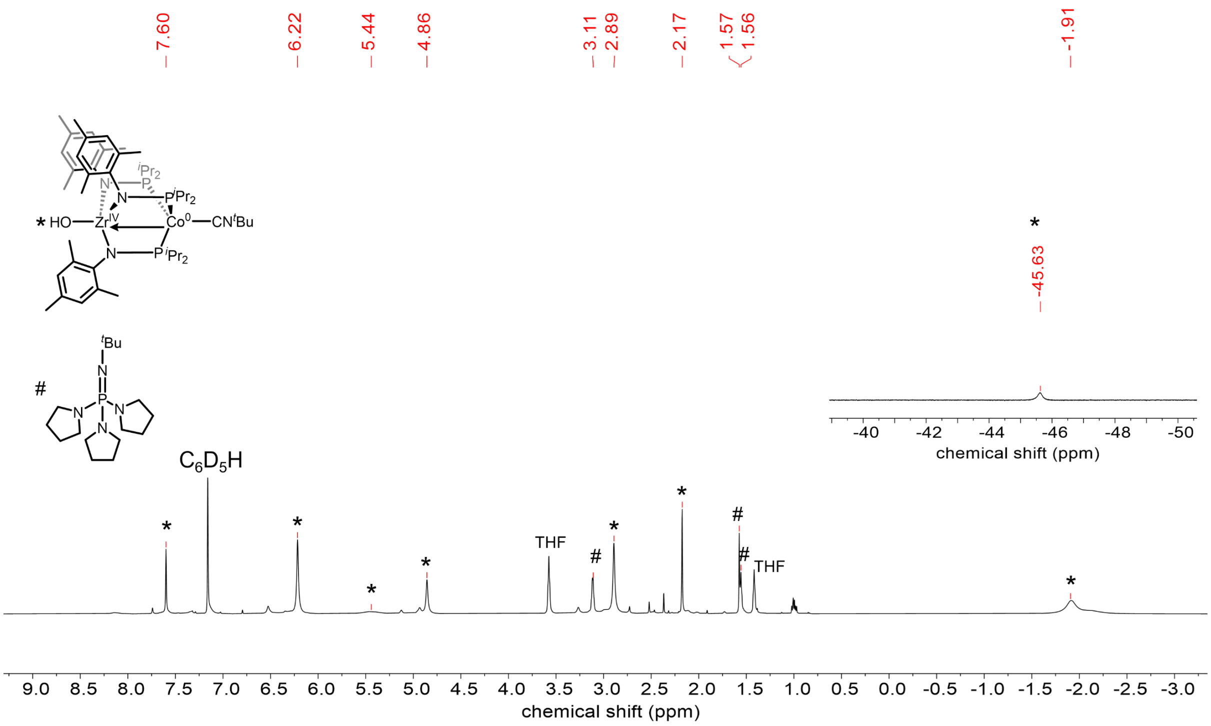

Figure S25. ^{1}H NMR spectrum (600 MHz, C_6D_6) of the crude reaction between **2**$^{-}$ and [^{t}BuHP$_1$pyrr][BPh_4] after 1 h of stirring.

pK_a [HO–Zr(MesNP^{i}Pr$_2$)$_3$CoCN^{t}Bu][BPh4] (1^{+}) = 21.7

5.3. O≡Zr(MesNP^{i}Pr$_2$)$_3$CoCN^{t}Bu (2) with ($Me_3Si)_2NH$

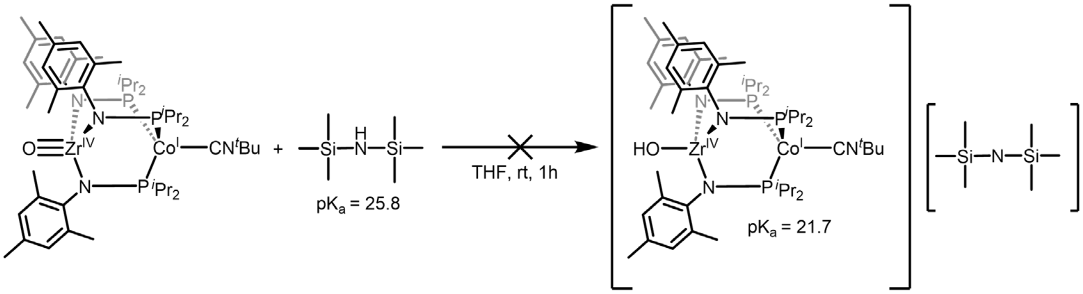

$(Me_3Si)_2NH$ (1.9 mg, 0.012 mmol) was added to a stirring solution of complex **2** (11.1 mg, 0.0111 mmol) in THF (~2 mL). There was no color change observed during the reaction; the solution remained green. The reaction mixture was allowed to stir at room temperature for 1 hour. The THF was removed under vacuum prior to ^{1}H NMR analysis.

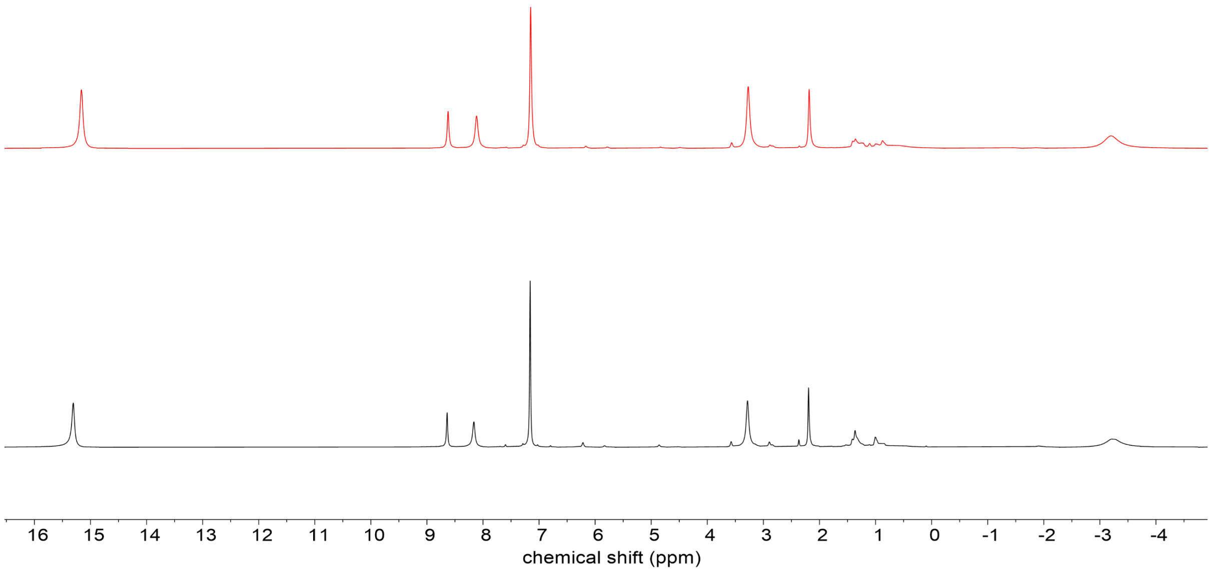

Figure S26. ^{1}H NMR spectra (400 MHz, C_6D_6) of **2** (top, red) and the crude reaction between **2** and $(Me_3Si)_2NH$ after 1 h of stirring (bottom, black).

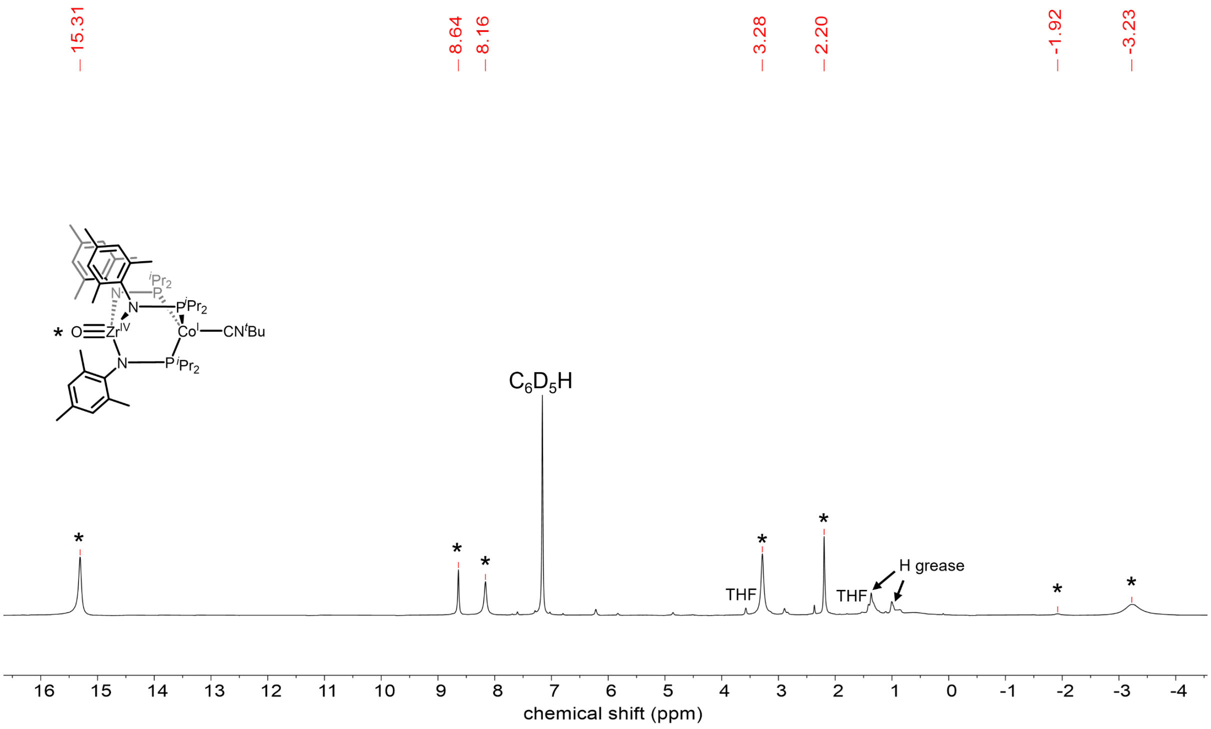

Figure S27. ^{1}H NMR spectrum (400 MHz, C_6D_6) of the crude reaction between **2** and $(Me_3Si)_2NH$ after 1 h of stirring.

5.4. O≡Zr(MesNP^{i}Pr$_2$)$_3$CoCN^{t}Bu (2) with [HNEt$_3$][BPh$_4$]

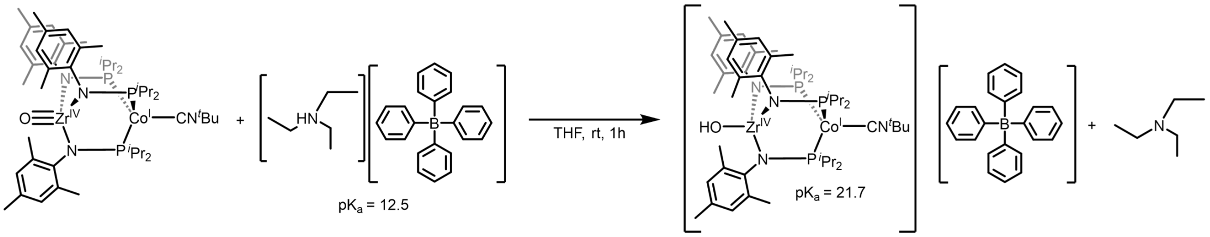

Triethylammonium tetraphenylborate (5.8 mg, 0.014 mmol) was dissolved in THF (~2 mL) and added to a stirring solution of complex **2** (12.0 mg, 0.0120 mmol) in THF (~2 mL). The reaction mixture went from bright green to a green-yellow color. The reaction mixture was allowed to stir at room temperature for 1 hour. The THF was removed under vacuum prior to ^{1}H NMR analysis. The reaction product matches the previously synthesized cationic hydroxide (**1$^+$**) product.[2]

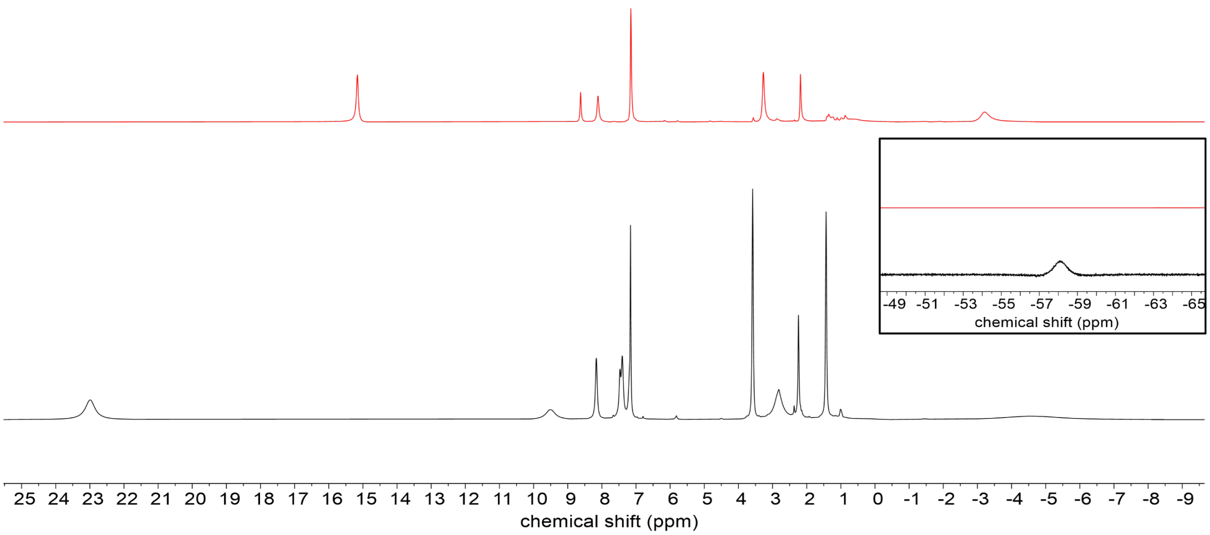

Figure S28. ^{1}H NMR spectra (400 MHz, C_6D_6) of **2** (top, red) and the crude reaction between **2** and triethylammonium tetraphenylborate after 1 h of stirring (bottom, black). Inset shows the region containing the hydroxide proton of $\mathbf{1}^{+}$.

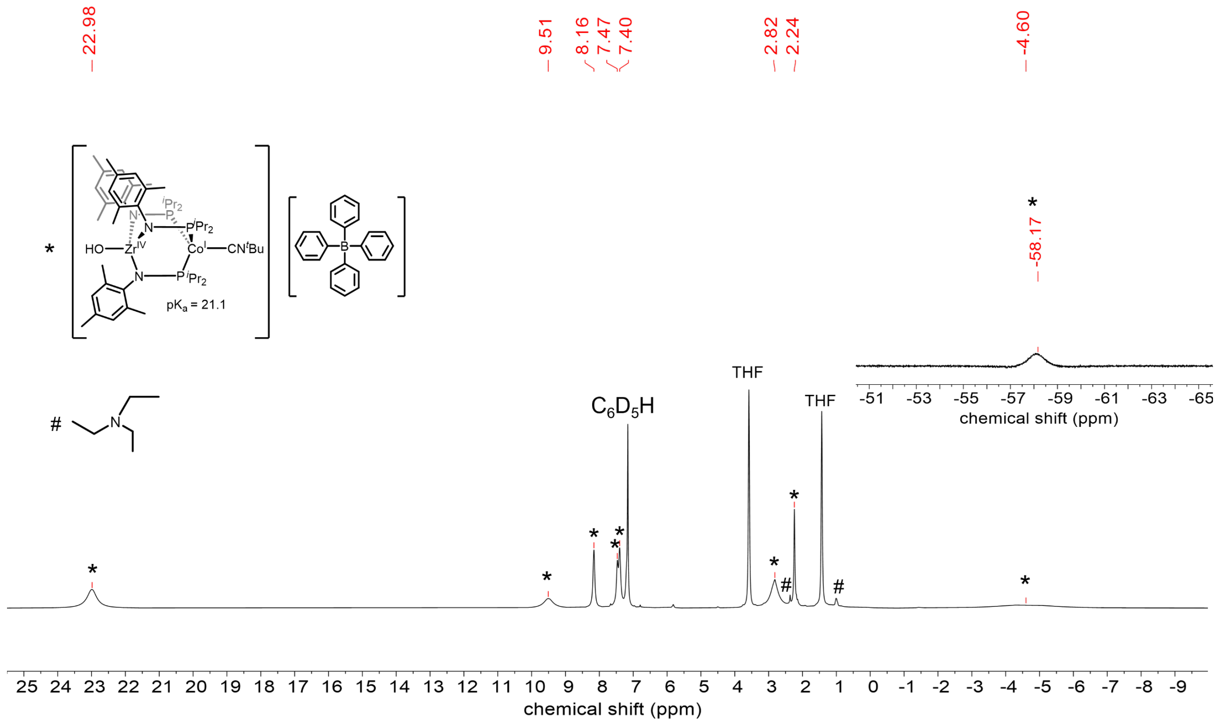

Figure S29. ^{1}H NMR spectrum (400 MHz, C_6D_6) of the crude reaction between **2** and triethylammonium tetraphenylborate after 1 h of stirring.

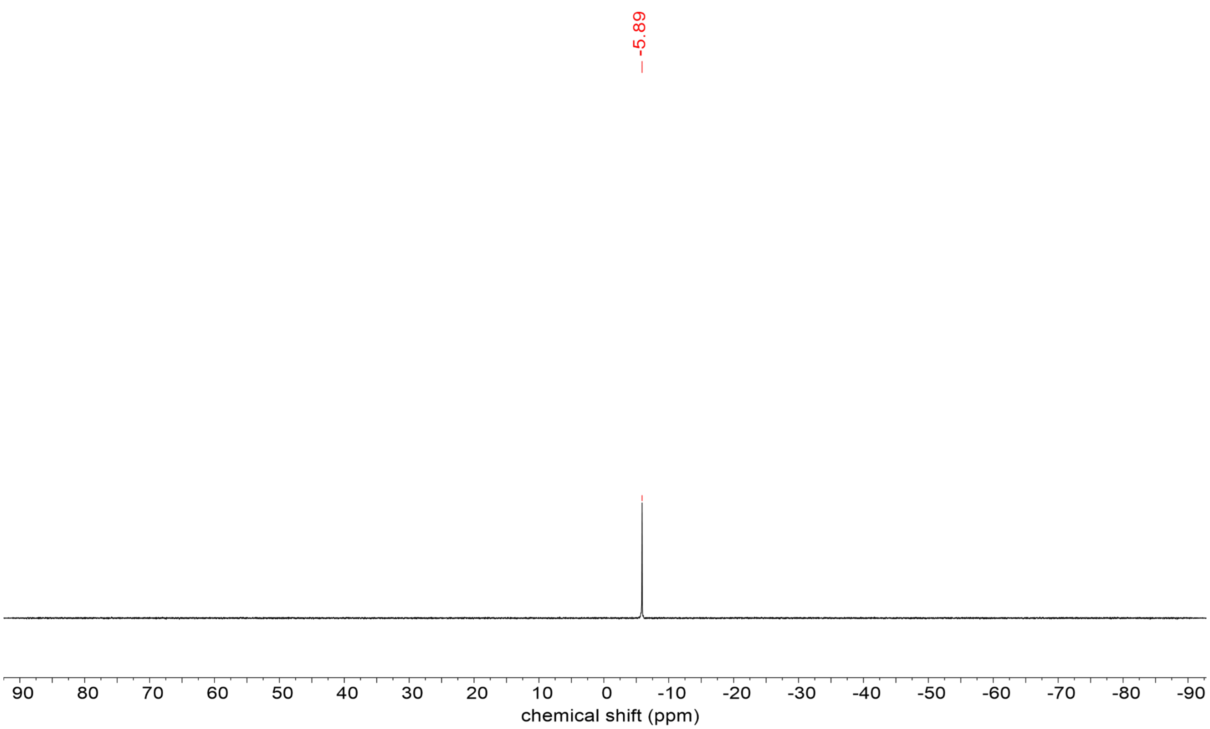

Figure S30. ^{11}B NMR spectrum (128 MHz, C_6D_6) of the crude reaction between **2** and triethylammonium tetraphenylborate after 1 h of stirring.

6. Computational Details

6.1. General computational considerations

All calculations were performed in the ORCA 5.0.3 software package.[12,13] Calculations were performed using the *ω*B97X-D3 functional[14] using either the basis set def2-SVP or def2-TZVPP.[15–17] All geometry optimizations were performed at the *ω*B97X-D3/def2-SVP level using the Conductor-like Polarizable Continuum Method (CPCM, solvent = THF)[18] to account for solvent effects. Minima were confirmed by the absence of any imaginary frequencies less than -15 cm^{-1} using analytical frequency calculations. Solvated single point energies were calculated at the def2-TZVPP level using the optimized geometries obtained at the def2-SVP level. Final solvated free energies were obtained by combining the solvated stationary point energies at the def2-TZVPP level with thermochemical correction factors from the frequency calculation at the def2-SVP level.

6.2. Estimating $BDFE_{O\text{-}H}$ using a DFT-based BDFE calibration curve

A calibration curve was constructed using a series of known BDFE values to predict the $BDFE_{O\text{-}H}$ of complex **1**. Eight small molecule H-atom donors with known experimental BDFE values in THF[10,19] (see Table S1 below) were used to make the calibration curve. The calibration curve was obtained by plotting the experimental BDFE values in THF (see Figure S31 below) versus the $\Delta G_{R,1}$ values, calculated using DFT. $\Delta G_{R,1}$ values for X–H bond homolysis were determined using the following equations:

$$\mathbf{X - H \rightarrow X^{\bullet} + H^{\bullet}}$$

$$\Delta \mathbf{G_{BDFE}} = \Delta \mathbf{G_{rxn}} = (\mathbf{G_{X\bullet}} + \mathbf{G_{H\bullet}}) - \mathbf{G_{X-H}} = \Delta \mathbf{G_{R,1}} + \Delta \mathbf{G_{H\bullet}}$$

where,

$$\Delta \mathbf{G_{R,1}} = \mathbf{G_{X\bullet}} - \mathbf{G_{X-H}}$$

Note that $\Delta G_{R,1}$ does not involve the calculation of the free energy of $H^{\bullet}$ (i.e. $G_{H\bullet}$) and the inaccuracy associated with its estimation. The geometries of X–H and $X^{\bullet}$ were optimized at the *ω*B97X-D3/def2-SVP level and their THF solvated Gibbs free energies were calculated at the *ω*B97X-D3/def2-TZVPP level as described in general considerations (*vide supra*).

Table S1. List of $H^{\bullet}$ donors with known experimental X-H BDFE values[10,19] and the DFT computed $\Delta G_{R,1}$ values.

H• donor	*Experimental X–H BDFE in THF (kcal/mol)*[10]	*$\Delta G_{R,1}$ calculated using DFT (kcal/mol)*
A	56.3	377.0
B	62.6	386.6
C	65.5	372.2
D	67.2	392.8
E	71.3	384.0
F	73.5	392.8
G	74.4	391.3
H	83.9[19*]	419.9
**1**	60.1**	370.4
H_2O-Zr(MesNP^{i}Pr$_2$)$_3$CoCN^{t}Bu	42.9**	334.8

* BDFE value calculated from the reported BDE value of 90 kcal/mol.
** Predicted value from the BDFE calibration curve (Figure S31).

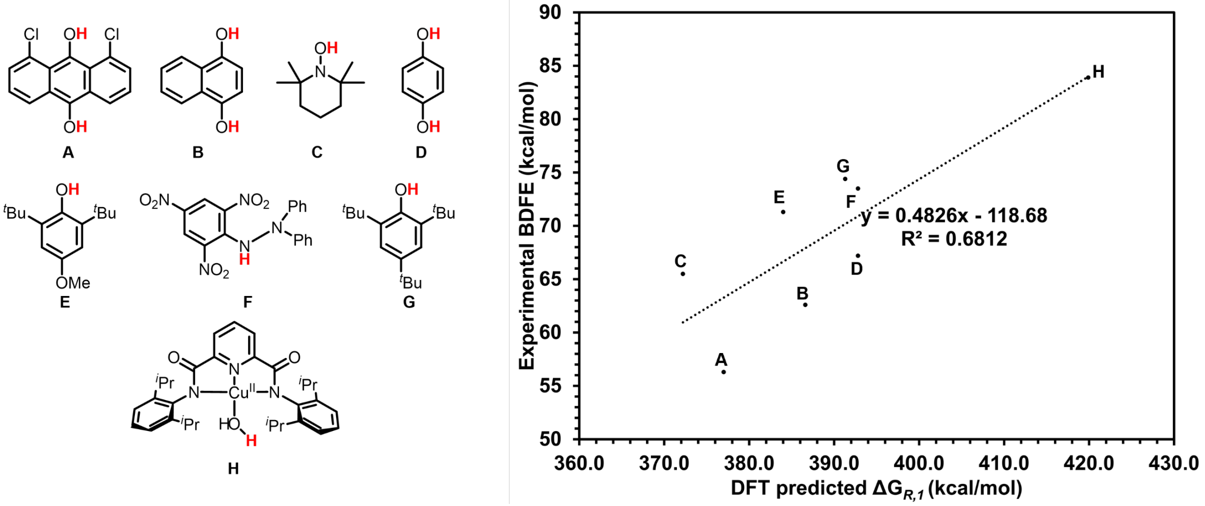

Figure S31. (**Left**) Structures of eight H$^{\bullet}$ donors used to construct the BDFE calibration curve in THF solvent. (**Right**): The BDFE calibration curve used to predict BDFE values, plotting experimental BDFE values in THF versus DFT-calculated $\Delta G_{R,1}$ values.

6.3. $BDFE_{O\text{-}H}$ sample calculation using the DFT BDFE calibration curve

$$\mathbf{BDFEsol(X-H) = 0.4826\Delta G_{R,1} - 118.68}\ \textbf{(eq. S3)}$$

$$\mathbf{BDFEsol(X-H) = 0.4826\ (370.4) - 118.68}$$

$$\mathbf{BDFEsol(X-H) = 60.1\ kcal/mol}$$

$$\mathbf{BDFEsol(X-H) = 60 \pm 4\ kcal/mol}$$

The calibration curve has a low correlation, as can be seen from the calibration curve in Figure S31 above, leading to the considerable error of +/- 4 kcal/mol in the calculated $BDFE_{O\text{-}H}$ value of **1**. For additional verification, pK_a values were also calculated using a second approach.

6.4. Estimating pK_a using a DFT-based pK_a calibration curve

A methodology similar to the construction of a calibration curve for X–H BDFE prediction was employed for the construction of a calibration curve for predicting pK_a values in THF. Nine organic acids with known pK_a values in THF[6,20–22] (see Table S2 below) were used to make the calibration curve. The calibration curve was obtained by plotting the experimental pK_a values in

THF (see Figure S32 below) versus the $\Delta G_{R,2}$ values, calculated using DFT. $\Delta G_{R,2}$ values for the H^+ dissociation reaction were determined using the following equations:

$$\mathbf{X - H \rightarrow X^- + H^+}$$

$$\Delta \mathbf{G_{acid}} = \Delta \mathbf{G_{rxn}} = (\mathbf{G_{X-}} + \mathbf{G_{H+}}) - \mathbf{G_{X-H}} = \Delta \mathbf{G_{R,2}} + \Delta \mathbf{G_{H+}}$$

where,

$$\Delta \mathbf{G_{R,2}} = \mathbf{G_{X-}} - \mathbf{G_{X-H}}$$

Note that as in the case of $\Delta G_{R,1}$, $\Delta G_{R,2}$ does not involve the Gibbs free energy of H^+ and is independent of the inaccuracy in estimating G_{H+}. Furthermore, since ΔG_{acid} is related to the pK_a, $\Delta G_{R,2}$ can be correlated to the pK_a of X–H and can be used to construct a calibration curve. The geometries of X**–**H and X^- were optimized at the *ω*B97X-D3/def2-SVP level and their THF solvated Gibbs free energies were calculated at the *ω*B97X-D3/def2-TZVPP level as described in general considerations (*vide supra*).

Table S2. List of acids with known experimental pK_a values in THF[6,20–22] and the DFT computed $\Delta G_{R,2}$ values.

Acid	***Experimental*** **p*K*a** ***in THF***	***ΔG**R,2* ***calculated using DFT (kcal/mol)***
I[6]	4.0	262.2
J[6]	5.2	264.4
K[6]	12.5	279.2
L[6]	13.5	280.4
M[6]	16.6	291.4
N[20]	20.8	294.2
O[20]	25.3	304.1
P[21]	32.2	330.3
Q[22]	34.5	334.2
**1**	27.9*	316.3
$\mathbf{1^+}$	21.8*	301.8

* Predicted value from the pK_a calibration curve (Figure S32).

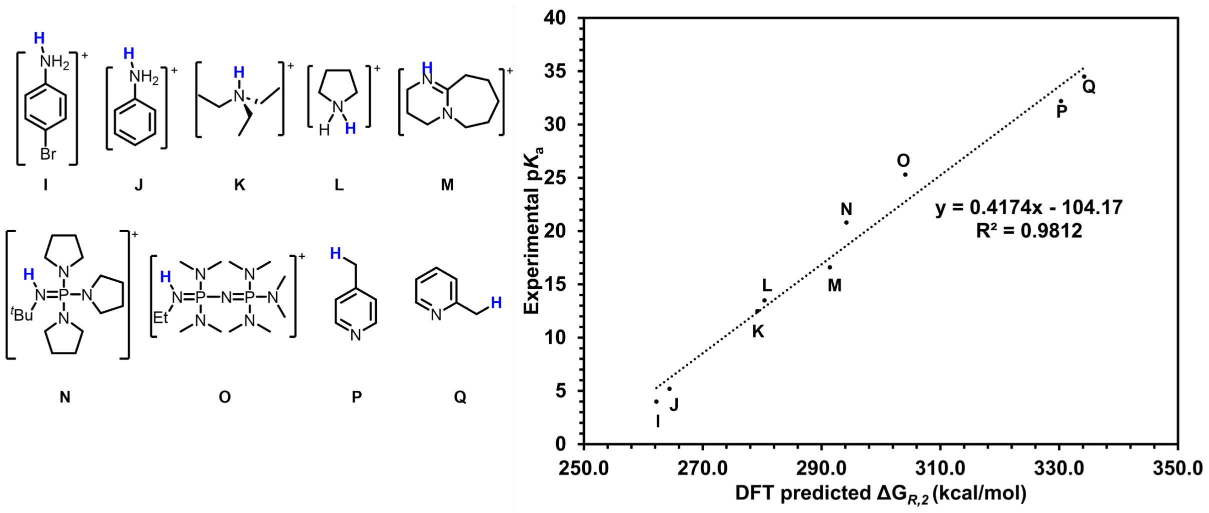

Figure S32. (**Left**) Structures of nine acids used to construct the pK_a calibration curve in THF solvent. (**Right**): The pK_a calibration curve used to predict pK_a values, plotting experimental pK_a values in THF versus DFT-calculated $\Delta G_{R,2}$ values.

6.5. pK_a sample calculation using the DFT pK_a calibration curve

$$\mathbf{pK_a(X-H) = 0.4174\Delta G_{R,2} - 104.17} \text{ (eq. S4)}$$

$$\mathbf{pK_a(HO-Zr/Co^0) = 0.4174(316.3) - 104.17}$$

$$\mathbf{pK_a(HO-Zr/Co^0) = 27.9}$$

$$\mathbf{pK_a(HO-Zr/Co^I) = 0.4174(301.8) - 104.17}$$

$$\mathbf{pK_a(HO-Zr/Co^I) = 21.8}$$

7. References

1 H. Zhang, G. P. Hatzis, C. E. Moore, D. A. Dickie, M. W. Bezpalko, B. M. Foxman and C. M. Thomas, *J. Am. Chem. Soc.*, 2019, 141, 9516–9520.
2 H. Zhang, G. P. Hatzis, D. A. Dickie, C. E. Moore and C. M. Thomas, *Chem. Sci.*, 2020, 11, 10729–10736.
3 V. W. Manner, T. F. Markle, J. H. Freudenthal, J. P. Roth and J. M. Mayer, *Chem Commun*, 2008, 256–258.
4 K. L. C. Grönberg, R. A. Henderson and K. E. Oglieve, *J. Chem. Soc. Dalton Trans.*, 1998, 3093–3104.
5 J. R. Dilworth, R. A. Henderson, P. Dahlstrom, T. Nicholson and J. A. Zubieta, *J Chem Soc Dalton Trans*, 1987, 529–540.
6 T. Rodima, I. Kaljurand, A. Pihl, V. Mäemets, I. Leito and I. A. Koppel, *J. Org. Chem.*, 2002, 67, 1873–1881.
7 C. F. Wise, R. G. Agarwal and J. M. Mayer, *J. Am. Chem. Soc.*, 2020, 142, 10681–10691.
8 F. G. Bordwell, J. Pei. Cheng and J. A. Harrelson, *J. Am. Chem. Soc.*, 1988, 110, 1229–1231.
9 F. G. Bordwell, J. Cheng, G. Z. Ji, A. V. Satish and X. Zhang, *J. Am. Chem. Soc.*, 1991, 113, 9790–9795.
10 R. G. Agarwal, S. C. Coste, B. D. Groff, A. M. Heuer, H. Noh, G. A. Parada, C. F. Wise, E. M. Nichols, J. J. Warren and J. M. Mayer, *Chem. Rev.*, 2022, 122, 1–49.

11 A. M. Smith and A. J. M. Miller, *Organometallics*, 2024, 43, 3163–3170.
12 F. Neese, *WIREs Comput. Mol. Sci.*, 2012, 2, 73–78.
13 F. Neese, *WIREs Comput. Mol. Sci.*, 2022, 12, e1606.
14 J.-D. Chai and M. Head-Gordon, *Phys. Chem. Chem. Phys.*, 2008, 10, 6615.
15 V. A. Rassolov, J. A. Pople, M. A. Ratner and T. L. Windus, *J. Chem. Phys.*, 1998, 109, 1223–1229.
16 F. Weigend and R. Ahlrichs, *Phys. Chem. Chem. Phys.*, 2005, 7, 3297.
17 J. Zheng, X. Xu and D. G. Truhlar, *Theor. Chem. Acc.*, 2011, 128, 295–305.
18 V. Barone and M. Cossi, *J. Phys. Chem. A*, 1998, 102, 1995–2001.
19 M. J. Bezdek, S. Guo and P. J. Chirik, *Science*, 2016, 354, 730–733.
20 I. Kaljurand, T. Rodima, A. Pihl, V. Mäemets, I. Leito, I. A. Koppel and M. Mishima, *J. Org. Chem.*, 2003, 68, 9988–9993.
21 R. R. Fraser, T. S. Mansour and S. Savard, *J. Org. Chem.*, 1985, 50, 3232–3234.
22 R. R. Fraser, M. Bresse and T. S. Mansour, *J. Chem. Soc. Chem. Commun.*, 1983, 620.
